# Transcriptomopathies of pre- and post-symptomatic frontotemporal dementia-like mice with TDP-43 depletion in forebrain neurons

**DOI:** 10.1186/s40478-019-0674-x

**Published:** 2019-03-29

**Authors:** Lien-Szu Wu, Wei-Cheng Cheng, Chia-Ying Chen, Ming-Che Wu, Yi-Chi Wang, Yu-Hsiang Tseng, Trees-Juen Chuang, C.-K. James Shen

**Affiliations:** 1 Institute of Molecular Biology, Academia Sinica, Nankang, Taipei, 115 Taiwan, Republic of China; 2 Genomics Research Center, Academia Sinica, Taipei, Taiwan; 3 Research Center for Environmental Changes, Academia Sinica, Taipei, Taiwan, Republic of China

**Keywords:** Circular RNAs/ frontotemporal lobar degeneration/ loss-of-function/ Mis-processing/TDP-43

## Abstract

TAR DNA-binding protein **(**TDP-43) is a ubiquitously expressed nuclear protein, which participates in a number of cellular processes and has been identified as the major pathological factor in amyotrophic lateral sclerosis (ALS) and frontotemporal lobar degeneration (FTLD). Here we constructed a conditional TDP-43 mouse with depletion of TDP-43 in the mouse forebrain and find that the mice exhibit a whole spectrum of age-dependent frontotemporal dementia-like behaviour abnormalities including perturbation of social behaviour, development of dementia-like behaviour, changes of activities of daily living, and memory loss at a later stage of life. These variations are accompanied with inflammation, neurodegeneration, and abnormal synaptic plasticity of the mouse CA1 neurons. Importantly, analysis of the cortical RNA transcripts of the conditional knockout mice at the pre−/post-symptomatic stages and the corresponding wild type mice reveals age-dependent alterations in the expression levels and RNA processing patterns of a set of genes closely associated with inflammation, social behaviour, synaptic plasticity, and neuron survival. This study not only supports the scenario that loss-of-function of TDP-43 in mice may recapitulate key behaviour features of the FTLD diseases, but also provides a list of TDP-43 target genes/transcript isoforms useful for future therapeutic research.

**Electronic supplementary material:**

The online version of this article (10.1186/s40478-019-0674-x) contains supplementary material, which is available to authorized users.

## Introduction

Frontotemporal lobar degeneration (FTLD) and amyotrophic lateral sclerosis (ALS) are both incurable and rapidly progressive neurodegenerative diseases of the central nerves system, and they have overlapping spectra of pathogenic features [75]. While patients with FTLD exhibit a range of progressive changes in language dysfunction, behavioural abnormality, personality change, memory deficit, or motor neuron dysfunction [71], muscle weakness and motor neuron degeneration are the predominant symptoms of ALS [76]. The cytoplasmic ubiquitinated inclusions (UBIs) consisting of relocated nuclear TDP-43 protein is a common pathological characteristic observed in 50% of FTLD (FTLD-TDP) and 95% of ALS (ALS-TDP) [4, 18, 47, 54].

TDP-43, or TAR DNA-binding protein-43 [55], encoded by the highly conserved *Tardbp* gene [82] is a RNA-binding protein involved in transcriptional repression, pre-mRNA splicing, and translation [1, 49, 62, 76, 83]. TDP-43 in the diseased cells of the patients’ brains of FTLD-TDP or spinal cords of ALS-TDP is characterized with abnormal ubiquitination, hyperphosphorylation, and enhanced cleavage to generate the 25 kDa and 35 kDa C-terminal fragments (TDP-25 and TDP-35) [4, 54]. Furthermore, TDP-43 is partially or completely cleared from the nuclei of neuronal and/ or glial cells containing cytosolic TDP-43 (+) UBIs [53].

Mouse models with transgenic overexpression of TDP-43, knock-out/ knock-down of *Tardbp* gene expression often serve as the biological system for exploring the physiological functions of TDP-43 and its pathogenic roles in neurodegeneration [75]*.* Most of the transgenic TDP-43 mouse lines overexpress human TDP-43, wild type or mutants, under the control of pan-neuronal promoter, and the resulting phenotypes appear to be primarily relevant to ALS [57, 63]. Studies have engineered the mice to overexpress wild-type TDP-43 or induced depletion of TDP-43 in the forebrain region, which sufficed to cause neurodegeneration of brain [33, 43, 78]. However, the pathological features of most of these various mouse models do not follow a pattern of adult-onset diseases. Furthermore, the analysis of their behavioural deficits has been predominantly based on motor function or Alzheimer disease-related tests [75]. On the other hand, FTLD patients with behavioural abnormalities (behavioural variant frontotemporal dementia, bvFTLD) present predominantly with persistent changes in behaviour and social functioning, which manifest in disinhibition, apathy, altered food preferences and executive deficits. Also, impairment of motor function and hippocampal-dependent learning/memory are rare in early stage bvFTLD [61].

Moreover, the relative contributions of loss-of-function and gain-of-cytotoxicity to the neurodegeneration in FTLD-TDP or ALS-TDP remain to be better defined [44, 45, 47, 76, 83]. The physiological functions of TDP-43 in different mammalian tissues also await further investigation. Our previous results have shown that TDP-43 is important for early mouse embryo development [88] and that loss-of-TDP-43 function in spinal motor neurons can generate many of the ALS-TDP phenotypes [89]. To explore the normal physiological function of TDP-43 and examine whether depletion of TDP-43 expression in brain could cause the neurodegeneration in FTLD-TDP, we have utilized the *Tardbp*^lx^ mouse line [88] and generated conditional knockout mice (TDP-43 cKO) with forebrain-specific depletion of TDP-43. We find that these mice exhibit a range of pathological phenotypes in striking similarity to FTLD. We further generate high-throughput RNA sequencing (RNA-seq) data from pre- and post-symptomatic TDP-43 cKO mice and the corresponding wild type mice, and show that some of these pathological phenotypes correlate well with specific changes of the gene expression profile in the forebrain upon depletion of TDP-43.

## Results

### Generation of mouse lines with *αCaMKII* promoter-directed depletion of forebrain TDP-43

**Fig. 1:**
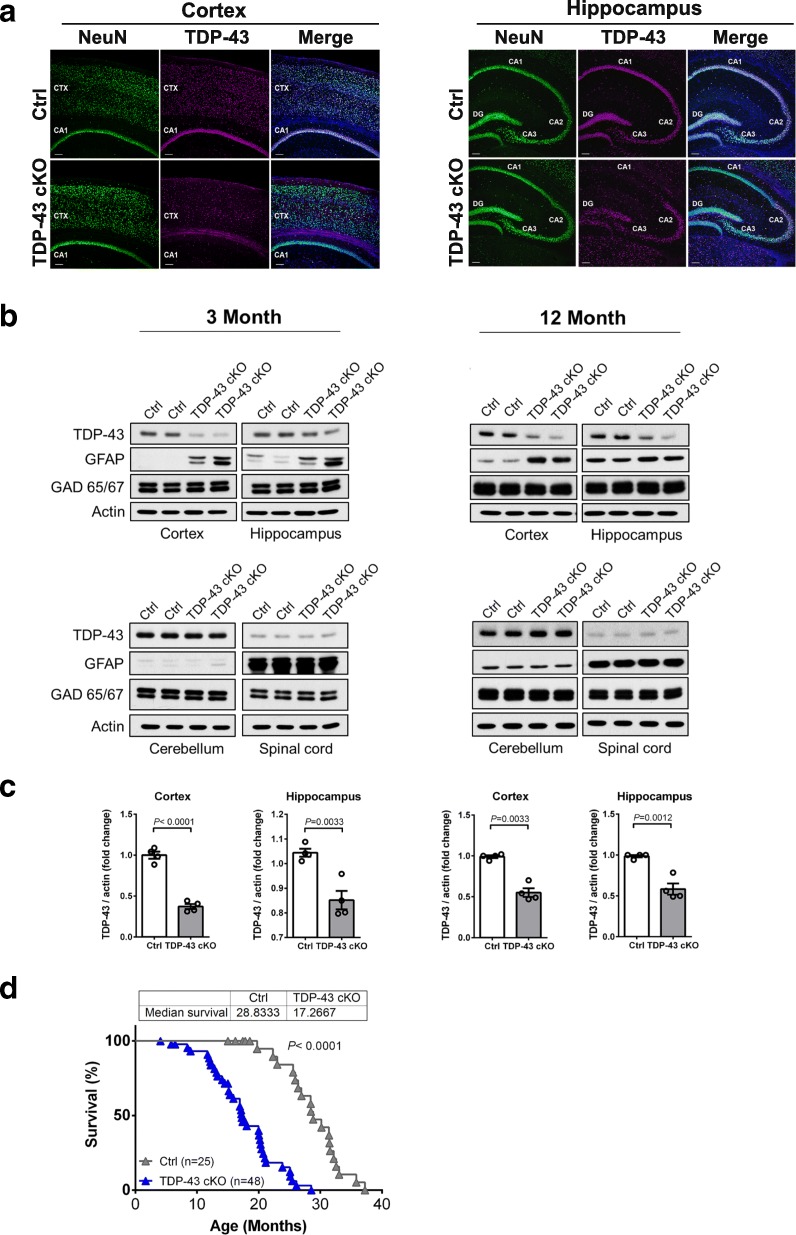
A mouse model (TDP-43 cKO) with *αCaMKII* promoter-directed depletion of TDP-43 in forebrain neurons. **a** Immunofluorescence staining analysis of brain sections from a cohort of 2-month-old Ctrl and TDP-43 cKO mice (TDP-43, magenta; NeuN, green; DAPI, blue). Cortex, CTX; cornu ammonis area, CA; dentate gyrus, DG. Scale bar represents 100 μm. **b** Western blotting analysis of the relative expression levels of TDP-43 and GFAP proteins in different tissues including cortex, hippocampus, cerebellum, and spinal cord of 3- and 12-month-old TDP-43 cKO mice and Ctrls. Note that ~ 50% reduction of TDP-43 protein level in the hippocampus and ~ 60% reduction of TDP-43 protein level in the cortex, but not in the cerebellum, and spinal cord in TDP-43 cKO mice. The Western blot patterns are exemplified in **b**, and the results of statistical analysis by unpaired student’s t test are shown in **c** *P <* 0.05 was considered significant. Data are represented as the average of 4 mice per group, with error bars reported as SEM. **d** Kaplan-Meyer survival curves of TDP-43 cKO mice and the Ctrls. Note the significantly shortened lifespan of TDP-43 cKO mice (*P* < 0.0001, log-rank Mantel-Cox test). The mean survival days are 28.8 months for the Ctrl mice and 17.2 months for the TDP-43 cKO mice

To understand the pathophysiological role of TDP-43 in adult brain, we generated conditional knock-out mice with forebrain-specific deletion of *Tardbp* gene by crossing *Tardbp* floxP mice with α*CaMKII*-Cre mice (T29–1 line) [88], the latter of which express α*CaMKII*-Cre only in neurons of the adult mouse brain [79]. Mice with *Tardbp*^*flox/flox*, Cre+^ alleles, referred to as TDP-43 cKO, were born at normal Mendelian ratios and appeared indistinguishable from their wild type littermate controls (*Tardbp*^*flox/flox*, Cre-^, referred to as Ctrl) at birth. As expected, immunohistochemistry analysis of the TDP-43 cKO mice at 2 months of age confirmed the deletion of *Tardbp* gene and consequent depletion of TDP-43 expression in the forebrain region, in particular in the CA1 pyramidal cell layer of the hippocampus (Fig. 1a and Additional file 1: Figure S2a). Western blot analysis of TDP-43 expression in the TDP-43 cKO mice at 3 and 12 months of age also supported that depletion of TDP-43 was restricted to the cortex and hippocampus (upper panels of Fig. 1b and c), but not in cerebellum and spinal cord (lower panels of Fig. 1b). 50% of TDP-43 cKO mice died around the age of 17 months, approximately 12 months shorter than the Ctrl mice (Fig. 1d). Collectively, the results in Fig. 1 demonstrate the successful establishment of a mouse model with postnatal depletion of TDP-43 in the forebrain and the shortened life span of TDP-43 cKO mice in comparison to their wild type littermate controls.

### Perturbation of social behaviour and development of dementia-like behaviour in TDP-43 cKO mice at the early stage of behaviour variations

**Fig. 2:**
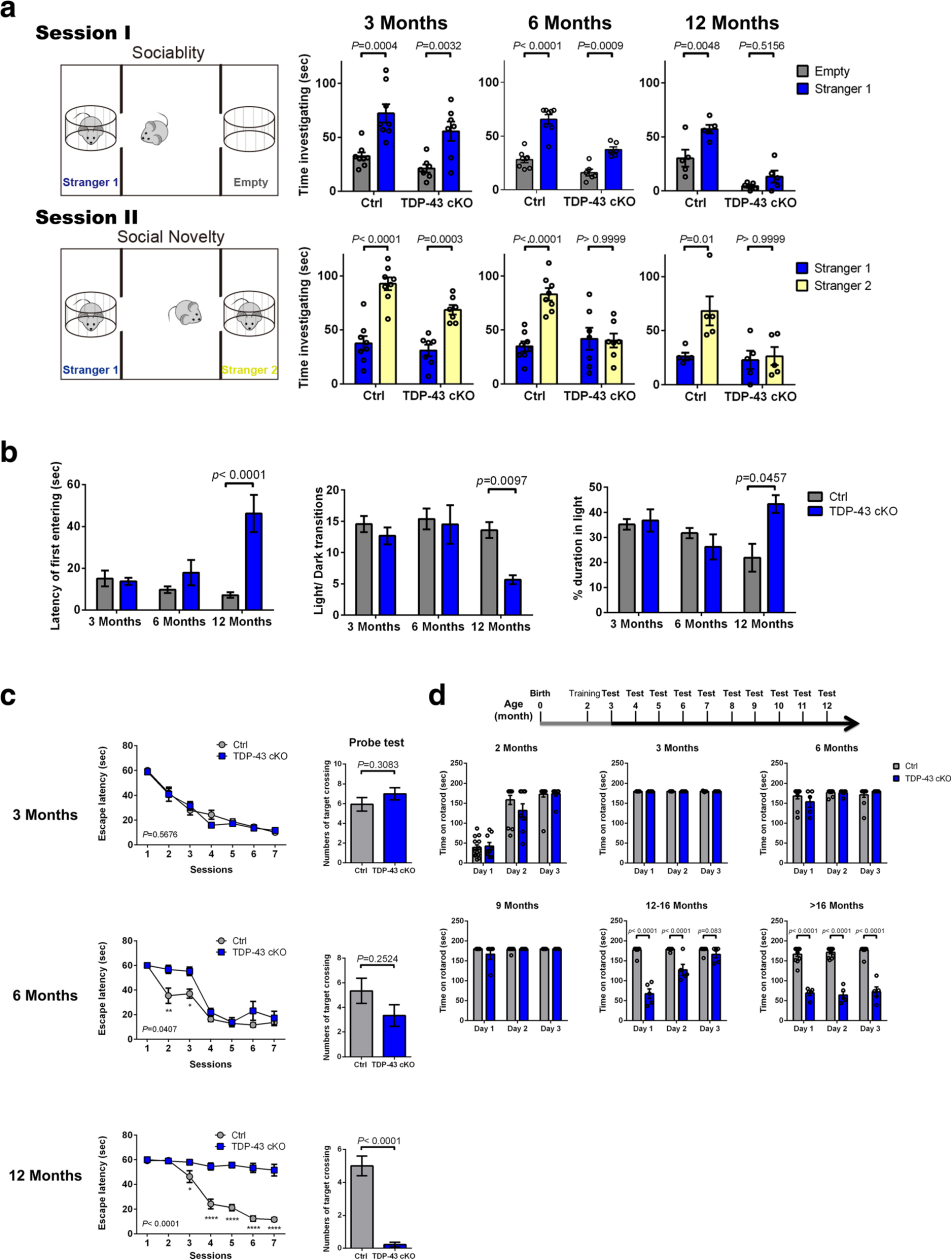
Progressively dismissing of social recognition and development of behaviour variants of the TDP-43 cKO mice. **a** For social interaction test (Session 1), the mean lengths of time (±SEM) the mouse spent in the chamber with the stranger (“Stranger 1”) and in the opposite chamber (“Empty”) are compared in the histograms. For social memory and novelty test (Session II), the mean durations of time (±SEM) in the chamber with the unfamiliar mouse from the sociability phase (“Stranger 1”) and in the opposite chamber with a new unfamiliar mouse (“Stranger 2”) are compared in the histograms. Statistical analysis was done by Two-way ANOVA (*N* = 8/group) with error bars reported as SEM, *P <* 0.05 was considered significant. **b** Light/ dark box test. The latencies of mouse entering the dark box for the first time, the light/dark transition periods, and durations of mouse in the light box were measured for mice of the ages of 3 months, 6 months, and 12 months, respectively, and statistical analysis was done by unpaired t test (*N* = 12/group) with error bars reported as SEM, *P <* 0.05 was considered significant. **c** Morris water maze tests. In the hidden platform test (left panels), the 12-month-old TDP-43 cKO mice had longer latencies to escape onto the hidden platform. Statistical analysis was done in curves by Two way ANOVA (N = 8/group) with error bars reported as SEM, *P <* 0.05 was considered significant. In the probe trial on the 8th session (right panels), the 12-month-old TDP-43 cKO mice traveled crossed the target, where the hidden platform was previously placed, significantly less times than the Ctrl mice. Statistical analysis was done in the histograms by unpaired t test with error bars reported as SEM. **d** Accelerated rotarod test. Ctrl and TDP-43 cKO mice were trained at the age of 2 months and then tested monthly on the accelerated rotarod (*N* = 11~14 in Ctrl group; *N* = 6~9 in TDP-43 cKO group). The histograms of latencies before falling showed that at 12 months of age, the TDP-43 cKO mice failed to memorize the rotarod running but re-learned right after the second day of training. At the end stage, e.g. older than 16 months, the TDP-43 cKO mice were unable to re-learn the rotarod running. Statistical analysis was done by unpaired t test with error bars reported as SEM, *P <* 0.05 was considered significant

TDP-43 has been identified as the major pathological protein in 50% of FTLD patients [80] and FTLD is characterized by a preponderance of abnormalities in social behaviour rather than memory, especially in the early stages of the disease [61]. Hence, we tested the social interaction behaviour of TDP-43 cKO mice by the three-chamber sociability and social novelty test [38] at 3, 6, and 12 months of age. In the test for social preference (session I), unlike the Ctrls, 12-month-old TDP-43 cKO mice showed no preference for their conspecific (stranger 1) over the object by spending similar time investigating the empty cage and stranger 1 (upper panels, Fig. 2a). In the test for preference of social novelty and social recognition (session II), either 6- or 12-month-old TDP-43 cKO mice failed to demonstrate a preference for unfamiliar mouse (stranger 2) compared with the familiar one (stranger 1) (lower panels, Fig. 2a). TDP-43 cKO mice exhibited a progressive decline in their capability of social interaction as observed among the bvFTLD patients.

The light/dark box test [20] was used to assess the anxiety-like behaviour [74] of the TDP-43 cKO mice. TDP-43 cKO mice at 12-month-old showed an increased latency in moving from the brightly lit area to the dark area, an increase in time spent in the light area, and markedly decreased crossings between light and dark area in comparison to the Ctrl mice (Fig. 2b). These data suggest that depletion of TDP-43 in mice lead to develop the dementia-like behaviour [43].

### Sequential impairment of learning/ memory capability and locomotor function in TDP-43 cKO mice at later stage of behaviour variations

FTLD patients usually did not display cognitive deficits until later stage of the disease [52]. We used the Morris Water Maze to examine the hippocampus-dependent learning/memory, including acquisition of spatial memory and long-term spatial memory [81] of TDP-43 cKO mice. As shown in Fig. 2c, TDP-43 cKO mice show severe learning/memory impairment at the age of 12 months (left panels of Fig. 2c). In the probe trial, only the 12-month-old TDP-43 cKO mice exhibited a significant smaller numbers of target platform crossings in comparison to the age-matched Ctrls (right panels of Fig. 2c). Overall, the progressive dementia of the TDP-43 cKO mice strongly suggests that the functional requirement of TDP-43 in learning/ memory at later stage of life [43].

Defective motor coordination developed in the late stages of a proportion of patients with FTLD [11]. We thus examined the locomotor activity of TDP-43 cKO mice using the accelerated rotarod tests. While no difference in motor performance could be found between Ctrl and TDP-43 cKO mice before the age of 12 months, older TDP-43 cKO mice exhibited reduced motor performance on the 1st day of test (Fig. 2d). Interestingly, their performance would become better on the 2nd day of test, and there was no difference between the TDP-43 cKO and Ctrl mice on the 3rd day of test (Fig. 2d). This result suggested that the impairment of rotarod performance of the TDP-43 cKO mice was mainly due to their memory loss. They barely remembered how to perform on the rotarod, but could re-learn after 1st day of test. However, while the motor deficiency was also observed after 16 months of age, it could not be reversed on the 2nd or 3rd day of test (Fig. 2d). The massive degeneration of cortex might result in the reduced motor performance after 16 months of age which the TDP-43 cKO mice loss their memory and learning ability to perform rotartod. Neverless, the above data show that in addition to severe memory loss developed after 12 months of age, TDP-43 cKO mice also exhibit motor dysfunction after the age of 16 months. This pattern is in interesting parallel to the sequential impairment of these two neuronal functions during FTLD pathogenesis [75].

### Progressively changes of nesting behaviour and eating habits in TDP-43 cKO mice

Beside cognitive dysfunction, patients with dementia including FTLD also exhibit decreased activities of daily living (ADL). We analyzed the changes of eating habits in TDP-43 cKO mice and found reduced food intake by the aged TDP-43 cKO mice (Additional file 1: Figure S1c) but not in younger ones (Additional file 1: Figure S1a and S1b). We also compared nest construction scores [21] between the Ctrl and TDP-43 cKO mice. There was a significant difference in the nesting behaviour between TDP-43 cKO and Ctrls at 12 months of age (Additional file 1: Figure S1d). Taken together, these results indicate that accompanying with the dementia phenotype, aged TDP-43 cKO mice also developed a progressive decrease of their ADL.

### Brain atrophy, neuronal loss, and neuronal degeneration of the TDP-43 cKO mice

**Fig. 3:**
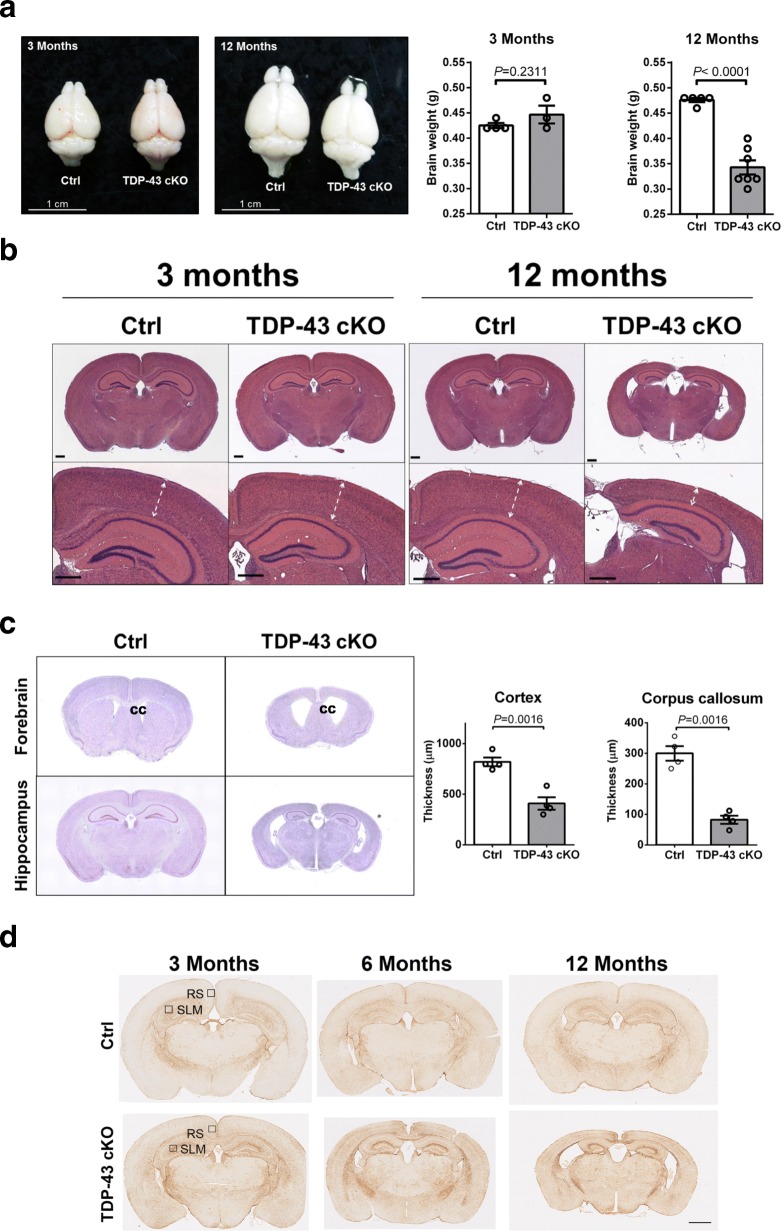
Brain atrophy and reactive astrocytosis in TDP-43 cKO mice. **a** Left, representative photo images of the brains of Ctrl and TDP-43 cKO mice at the ages of 3 months and 12 months. The total brain weights were also determined and compared in histograms on the right. *N* = 4–7 mice per group. Statistical analysis was done by unpaired t test with error bars reported as SEM, *P <* 0.05 was considered significant. **b** Representative histological images of the brain sections of 3- and 12-month-old TDP-43 cKO mice and their littermate Ctrls. More detailed characterizations are presented in Additional file 1: Figure S2. **c** Left panels, Nessie staining images of the forebrain and hippocampus sections from the Ctrl and TDP-43 cKO mice showing marked atrophy at corpus callosum (cc) and ventricle enlargement in the brain of 12-month-old TDP-43 cKO mice compared to Ctrls. The total cortex thickness and corpus callosum thickness were calculated by the length of cortex area and corpus callosum. N = 4 mice per group. Statistical analysis was done by unpaired t test with error bars reported as SEM, *P <* 0.05 was considered significant. **d** Immunohistochemistry staining with anti-GFAP revealing high numbers of GFAP-positive astrocytes in the retrosplenial cortex (RS) region of the cortex and stratum lacunosum-moleculare (SLM) region in the brain of TDP-43 cKO mice

Necropsy examination showed that the 12-month-old TDP-43 cKO mice, but not 3-month-old ones, had a significant reduction of the overall brain size and its weight when compared to age-matched Ctrls, which appeared to be mainly due to a decrease in the size of the cortical areas **(**Fig. 3a). In parallel, hematoxylin and eosin staining showed aberrant cellular patterns and layering in the cortex of 12-month-old TDP-43 cKO mice (Fig. 3b and Additional file 1: Figure S2). Also, the thickness of cerebral cortex was reduced and the size of the ventricles was enlarged when compared to the Ctrls (Fig. 3b and c). These results are indicated that depletion of TDP-43 causes the brain atrophy in mice [43].

**Fig. 4:**
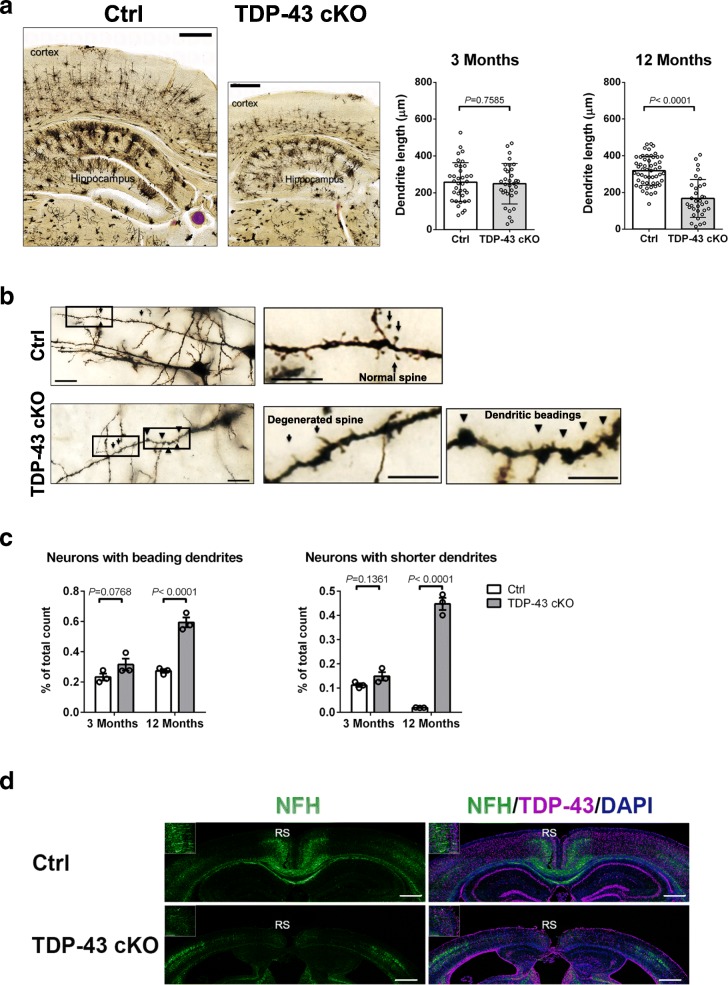
Dendritic alternations of neurons in the cortex of aged TDP-43 cKO mice. Golgi staining was used to visualize the neuronal dendrites in the cortex of 3- and 12-month-old TDP-43 cKO mice in comparison to the Ctrl mice. **a** Representative images of the Golgi-staining patterns in the left 2 panels show the obvious morphology changes and dendritic shortening of the cortical neuron of 12-month-old TDP-43 cKO mice in comparison to the Ctrl mice. Scale bar is 500 μm. Quantitative comparison of the dendritic lengths of cortical layer V neurons of TDP-43 cKO and Ctrl mice is shown in the right two diagrams. Note the significant reduction of the average dendritic length in 12-month-old TDP-43 cKO. Statistical analysis was done by unpaired t test with the error bars being SEM. *P <* 0.05 was considered significant. **b** Enlarged views of cortical layer V neurons. Representative images show the cortical dendrites with segmental beading dendrites (arrow heads) and degenerated spines (arrows), respectively. Scale bar is 20 μm. **c** Quantifications of the % of cortical layer V neurons with beading dendrites (left histogram) or shorter dendrites (right histogram). Statistically analyzed by unpaired t test with error bars reported as SEM, *P <* 0.05 was considered significant. Note the markedly increase of abnormal dendrites in TDP-43 cKO mice at the age of 12 months, but not 3 months. **d** Immunofluorescence staining with anti-neurofilament H (SMI-32) (green) showing a marked decrease of the neuron numbers in the cortical layer III/ V of the retrosplenial cortex (RS) of TDP-43 cKO mouse brain but not Ctrls. Enlarged views of RS region are shown in the upper left corner of each image

Massive neuronal degeneration was observed in the cortex and hippocampus of 12-month-old TDP-43 cKO mice (Additional file 1: Figure S2 b-d). In particular, Golgi staining showed that the average length of the dendrites on dendritic stems of the layer V neurons of 12-month-old TDP-43 cKO mice was shorter than that of the control group **(**Fig. 4a**)**. Morever, the numbers of neuron with the beading or shorter dendrites [48, 71, 72] were increased mainly in the layer V pyramidal neuron of TDP-43 cKO mice at the age of 12 months **(**Fig. 4b and c**)**. Immunofluorescense staining showed that the number of neurofilament H marker (SMI-32)-positive neurons in layer III/ V of retrosplenial cortex (RS), but not those in the somatosensory cortex, decreased in TDP-43 cKO mice (Fig. 4d). Taken together, there appears to be significant and progressive neuronal degeneration in TDP-43 cKO mice in a selective vulnerability manner, with distinct neuronal populations in different cortical layers compromised by the depletion of TDP-43.

### Chronic astrocytosis in the forebrain of TDP-43 cKO mice

We assessed the astrocyte response during the progressive cortical degeneration describe above. As shown, GFAP-positive astrocytes in the cortical layers and hippocampus of Ctrl mice of different ages were mainly found in the corpus callosum and rarely in the neuronal layers of the cortex (upper panels, Fig. 3d). Furthermore, their slim morphology suggested a resting state of the astrocytes (upper panels, Additional file 1: Figure S3a). In stark contrast, large numbers of tufted enlarged GFAP-positive astrocytes were found in the retrosplenial cortex (RS) and in the stratum lacunosum-moleculare (SLM) region of the hippocampus of TDP-43 cKO mice (lower panels, Fig. 3d and Additional file 1: Figure S3a) and they progressively increased during aging (Additional file 1: Figure S3a and b), coinciding with the progressive thinning of the cortex exemplified in Fig. 3b. However, no microglia activation was observed at all stages of the Ctrl and TDP-43 cKO mice analyzed (Additional file 1: Figure S3c). These results indicated that progressive astrogliosis, but not microgliosis was found in TDP-43 cKO mice which reflecting the neuropathological changes. Taken together, depletion of TDP-43 in the forebrain neurons resulted in a substantial and persisting activation of the astrocytes.

### Impaired synaptic plasticity in TDP-43 cKO mice

TDP-43 has been shown to be a modulator of synaptic plasticity in transgenic mouse models of ALS and FTLD [30]. We investigated whether depletion of TDP-43 indeed affected the synaptic plasticity by examining the Schaeffecr collateral pathway of TDP-43 cKO mice for synaptic plasticity, long-term potentiation (LTP) and long-term depression (LTD). As shown in Additional file 1: Figure S4, the magnitude of long-term potentiation (LTP) and long-term depression (LTD) in the hippocampal slices from 2-month-old TDP-43 cKO mice was unaffected (Additional file 1: Figure S4a and c). At the age of 12 months, LTP in the hippocampus of TDP-43 cKO mice were significantly lower than the Ctrl mice **(**Additional file 1: Figure S4b and c**)**. Alltogether, these results show that depletion of TDP-43 in the forebrain neurons affects the synaptic functions.

### Genome-wide analysis of the neocortex transcriptomes of pre- and post-symptomatic TDP-43 cKO mice in comparison to ctrl mice

**Fig. 5:**
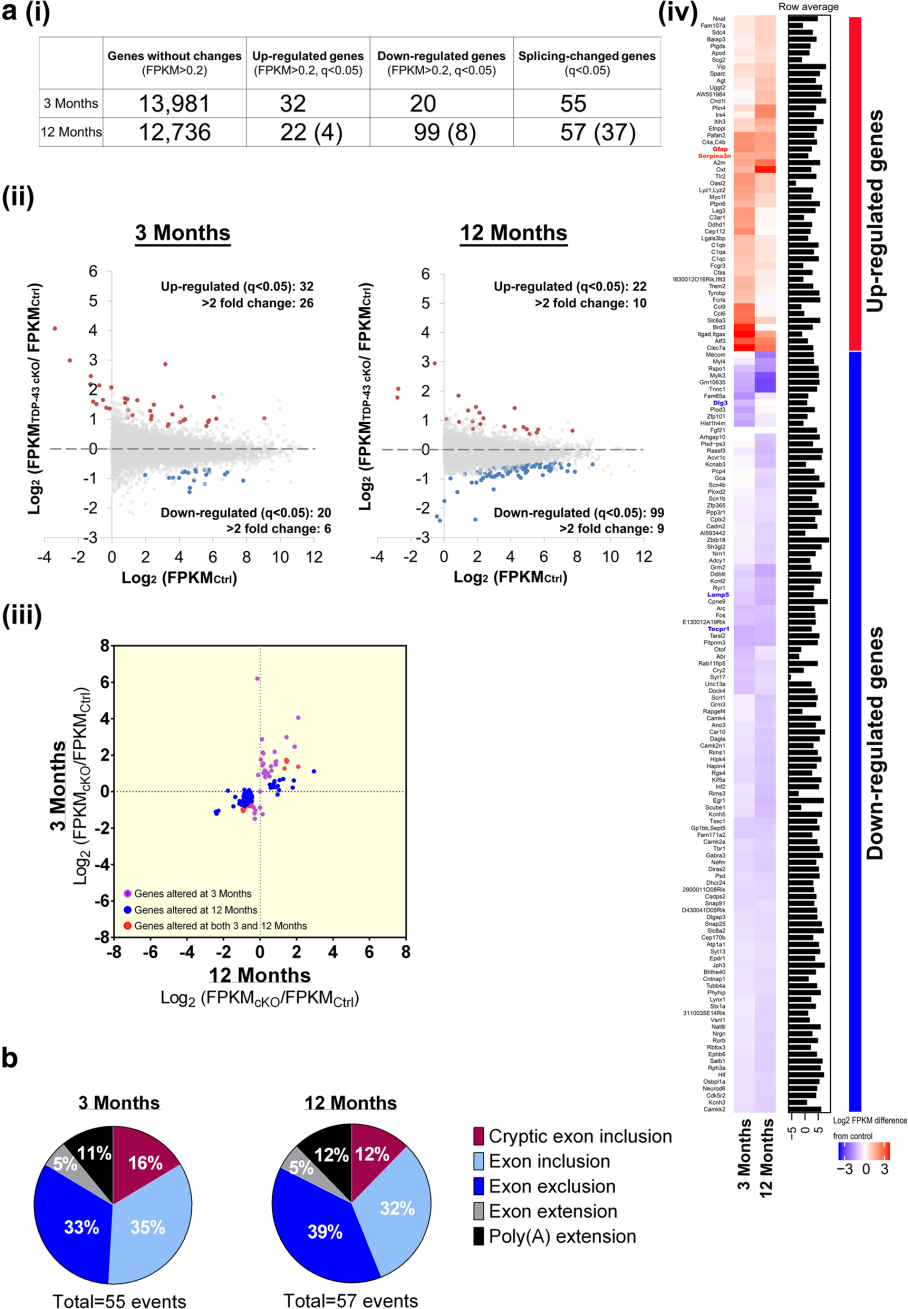
Analysis of the impact of mRNA transcriptomes in neocortex of 3- or 12-month-old TDP-43 cKO mice**. a(i),** The numbers of the unchanged, up-regulated, down-regulated, and splicing-changed genes, respectively, in the neocortex of 3- or 12-month-old TDP-43 cKO mice in comparison to the Ctrls. Only gene expression changed with FPKM> 0.2 were considered in the analysis. The numbers in the parentheses are the numbers of the differentially expressed genes in the neocortex of TDP-43 cKO mice at both the ages of 3 months and 12 months in comparison to the Ctrls. (**ii),** Scatter plot showing log_2_ fold change of neocortex gene expression of 3-month-old (left panel) and 12-month-old (right panel) TDP-43 cKO mice and control mice, respectively. The red, blue, and gray dots indicate up-regulated (q < 0.05), down-regulated (q < 0.05), and non-significantly changed genes, respectively, in the neocortex of TDP-43 cKO mice. Each dot represents the mean value of data from analysis of triplicate neocortex sample sets. (**iii),** Scatter plot showing the correlation of log_2_ fold change of genes altered in the neocortex of TDP-43 cKO mice at 12 months (x axis) and 3 months (y axis) of age. The purple and blue dots indicate neocortex genes the expression levels of which are altered only in 3-month-old TDP-43 cKO mice (40 genes) and only in 12-month-old TDP-43 cKO mice (109 genes), respectively. The 12 neocortex genes deregulated in TDP-43 cKO mice at both ages are indicated by the red dots (12 genes). (**iv),** Heat map representation of the expression patterns of the 161 (40 + 109 + 12) genes de-regulated in the neocortex of TDP-43 cKO mice. The expression levels of up-regulated and down-regulated genes are highlighted with different red and blue colors, respectively, in the 2 columns, with the individual gene names indicated on the side of the columns. **b**, Cortical RNAs of TDP-43 cKO and Ctrl mice at the age of 3 months or 12 months were analyzed by Cufflink/MISO as described. The percentages (%) of the processing alternations, i.e. alternative uses of poly-A sites (“poly (A) extension”), extensions of conserved exons (“Exon extension”), inclusions of conserved exons (“Exon inclusion”), exclusion of conserved exons (“Exon exclusion”) and inclusion of cryptic exons (“Cryptic exon inclusion”), are shown

Transcripts exhibiting alternations of their expression levels or splicing patterns in TDP-43 depleted cells other than cortex have been identified before by RNA-seq analysis [5, 37, 58]. However, a transcriptome-wide analysis of the relationship between TDP-43 targeted RNAs and TDP-43 regulated behaviour/phenotypes was lacking. We used paired-end deep sequencing **(see** **Materials and Methods****)** to examine the gene expression profiles in the neocortex (the region indicated in the left part of Additional file 1: Figure S2a) of TDP-43 cKO mice and their corresponding littermate controls at both the pre-symptomatic (3-month) and post-symptomatic (12-month) stages. The differential expression analysis (see Materials and Methods) revealed 52 and 121 up-/down-regulated genes at the ages of 3 months and 12 months, respectively **(**Fig. 5a-(i)). Interestingly, the number of the down-regulated genes was much greater in 12-month-old TDP-43 cKO mice than in 3-month-old cKO ones **(**Fig. 5a-(ii)), in correlation with the progression of the FTLD-like pathological phenotypes of the mutant mice **(**Fig. 2-4**)**.

Significantly, most of the transcripts with increased abundance in TDP-43 cKO mice associated with activation of astrocytes, whereas the majority of transcripts with decreased abundance were associated with calcium signaling and synaptic transmission—likely reflecting the astrocytosis and synaptic transmision deficit that progressively occurred with aging in TDP-43 cKO cortex **(**Table 1**)**. Consistent with the correlation of these up/down regulated cortex genes with the age-dependent pathogenesis of TDP-43 cKO mice, most of the gene expression differences between the TDP-43 cKO cortex and Ctrl cotex became greater magnified as the mice aged (Fig. 5a-(i) and 5a-(iv)).

**Table 1:** The genes with altered mRNA levels in the neocortex of 3-month and 12-month-old TDP-43 cKO mice in comparison to the Ctrls are listed

Gene symbol	Name	RefSeq ID	Log2(TDP-43 cKO/ Ctrl)		Significant (q<0.05)		FTLD patient	White et al. [86]				Other references
			3 Months	12 months	3 Months	12 months		5 Months frontal cortex		20 Months frontal cortex		
								Common	MB-	Common	MB-	
Rabggtb	Rab geranylgeranyltransferase beta subunit	NM_011231	6.21	-0.14	●							
Clec7a	C-type lectin domain containing 7A	NM_020008	4.06	2.09	●		(Chen-Plotkin et al. [17])					
Itgad	Integrin alpha-D	NM_001029872	2.99	1.45	●							
Brd3	bromodomain containing 3	NM_023336	2.88	0.11	●							
Atf3	activating transcription factor 3	NM_007498	2.47	1.90	●							
Slc6a3	solute carrier family 6 member 3	NM_010020	2.19	0.80	●							
Ccl9	chemokine (C-C motif) ligand 6	NM_011338	2.15	0.15	●							
Ccl6	chemokine (C-C motif) ligand 9	NM_009139	2.09	0.18	●							
Cep112	centrosomal protein 112	NM_029586	1.76	0.04	●							
Pafah2	platelet-activating factor acetylhydrolase 2	NM_001285872	1.69	1.50	●							
Tlr2	toll like receptor 2	NP_036035	1.66	0.88	●							
Oasl2	2'-5' oligoadenylate synthetase-like 2	NM_011854	1.61	0.85	●							
Lag3	lymphocyte-activation gene 3	NM_008479	1.55	0.17	●			●				
C3ar1	complement C3a receptor 1	NP_033909	1.52	0.18	●		(Chen-Plotkin et al. [17])					
Lyz1,Lyz2	lysozyme 1	NM_017372	1.48	0.86	●							
Ddhd1	DDHD domain containing 1	NM_176845	1.44	0.13	●							
Myo1f	myosin IF	NM_053214	1.39	0.60	●					●		
Trem2	triggering receptor expressed on myeloid cells 2	NM_001272078	1.16	0.25	●							(Polymenidou et al. [58])
Ptpn6	protein tyrosine phosphatase, non-receptor type 6	NM_001077705	1.15	0.63	●					●		
Ifit3b	interferon-induced protein with tetratricopeptide repeats 3B	NM_001005858	1.11	0.31	●							(Polymenidou et al. [58])
C1qa	complement component 1, q subcomponent, alpha polypeptide	NM_007572	1.02	0.42	●							(Polymenidou et al. [58])
Tyrobp	TYRO protein tyrosine kinase binding protein	NM_011662	1.01	0.19	●							(Polymenidou et al. [58])
Lgals3bp	lectin, galactoside-binding, soluble, 3 binding protein	NM_011150	0.99	0.38	●		(Chen-Plotkin et al. [17])					
C1qb	complement component 1, q subcomponent, beta polypeptide	NM_009777	0.96	0.41	●					●		(Polymenidou et al. [58])
C1qc	complement component 1, q subcomponent, C chain	NM_007574	0.90	0.39	●					●		
Fcrls	Fc receptor-like S, scavenger receptor	NM_030707	0.91	-0.11	●			●				
Fcgr3	Fc receptor, IgG, low affinity III	NM_010188	0.83	0.53	●					●		(Polymenidou et al. [58])
Ctss	cathepsin S	NM_001267695	0.79	0.28	●							(Polymenidou et al. [58])
Ripor1	Rho family interacting cell polarization regulator 1	NM_001081241	-1.48	-0.28	●							
Plod3	procollagen-lysine, 2-oxoglutarate 5-dioxygenase 3	NM_011962	-1.23	0.14	●					●		
Hist1h4m	histone cluster 1, H4m	NM_175654	-1.18	-0.30	●							
Zfp101	zinc finger protein 101	NM_009542	-1.02	-0.26	●					●		
Dlg3	discs large MAGUK scaffold protein 3	NM_007863	-0.87	0.00	●		(Chen-Plotkin et al. [17])					(LaClair et al. [43])
Otof	otoferlin	NM_001100395	-0.86	-0.34	●				● (up)			
Dock4	dedicator of cytokinesis 4	NM_172803	-0.79	-0.52	●					●		(Polymenidou et al. [58])
Abr	active BCR-related gene	NM_001291186	-0.79	-0.41	●							(LaClair et al. [43])
Syt17	synaptotagmin XVII	NM_138649	-0.70	-0.48	●							
Unc13a	unc-13 homolog A (C. elegans)	NP_001025044	-0.77	-0.48	●							(Polymenidou et al. [58])
Rab11fip5	RAB11 family interacting protein 5 (class I)	NM_001003955	-0.69	-0.58	●							
Fgf21	fibroblast growth factor 21	NM_020013	0.00	0.00	●							
Gfap	glial fibrillary acidic protein	NM_001131020	1.74	1.43	●	●	(Chen-Plotkin et al. [17])					(Polymenidou et al. [58])
Serpina3n	serine (or cysteine) peptidase inhibitor, clade A, member 3N	NP_035588	1.27	1.34	●	●						
C4a,C4b	complement component 4a/b	NM_011413	1.64	1.46	●	●	(Chen-Plotkin et al. [17])					
A2m	alpha-2-macroglobulin	NM_175628	1.37	2.09	●	●						
Tecpr1	tectonin beta-propeller repeat containing 1	NP_081686	-1.06	-0.93	●	●						(LaClair et al. [43])
Tarsl2	threonyl-tRNA synthetase-like 2	NM_172310	-1.00	-0.90	●	●						
Pitpnm3	PITPNM family member 3	NM_001024927	-0.97	-0.96	●	●			● (up)		● (up)	(LaClair et al. [43])
Epop	elongin BC and polycomb repressive complex 2 associated protein	NM_175332	-0.80	-0.90	●	●						
Arc	activity regulated cytoskeletal-associated protein	NM_001276684	-0.84	-0.80	●	●	(Chen-Plotkin et al. [17])					(Polymenidou et al. [58])
Cry2	cryptochrome 2 (photolyase-like)	NM_009963	-0.78	-0.58	●	●						
Lamp5	lysosomal-associated membrane protein family, member 5	NM_029530	-0.71	-0.96	●	●						
Fam171a2	family with sequence similarity 171, member A2	NM_199200	-0.70	-0.65	●	●		●				
Oxt	oxytocin/neurophysin I prepropeptide	NM_011025	1.11	2.95		●						
Plin4	perilipin 4	NM_020568	0.61	1.85		●		●				
Irs4	insulin receptor substrate 4	NP_034702	0.22	1.79		●						
Itih3	inter-alpha trypsin inhibitor, heavy chain 3	NM_008407	0.69	1.27		●						
Etnppl	ethanolamine phosphate phospholyase	NM_001163587	0.63	1.07		●						
AW551984	expressed sequence AW551984	NM_001199556	0.08	1.04		●						
Agt	angiotensinogen (serpin peptidase inhibitor, clade A, member 8)	NM_007428	0.31	0.97		●				●		
Chd1l	chromodomain helicase DNA binding protein 1-like	NM_026539	-0.16	0.96		●						
Uggt2	UDP-glucose glycoprotein glucosyltransferase 2	NM_001081252	0.26	0.87		●				●		
Vip	vasoactive intestinal polypeptide	NM_001313969	0.57	0.79		●						
Sparc	secreted acidic cysteine rich glycoprotein	NM_001290817	0.48	0.77		●	(Chen-Plotkin et al. [17])	●			● (down)	
Baiap3	BAI1-associated protein 3	NM_001163270	0.24	0.72		●						
Nnat	neuronatin	NM_001291128	0.37	0.70		●						
Fam107a	family with sequence similarity 107, member A	NM_183187	0.38	0.69		●						
Sdc4	syndecan 4	NM_011521	0.33	0.68		●		●				(Polymenidou et al. [58])
Ptgds	prostaglandin D2 synthase (brain)	NM_008963	0.27	0.65		●	(Chen-Plotkin et al. [17])					
Scg2	secretogranin II	NM_001310680	0.21	0.58		●	(Chen-Plotkin et al. [17])					
Apod	apolipoprotein D	NM_001301353	0.38	0.54		●	(Chen-Plotkin et al. [17])					(Polymenidou et al. [58])
Tnnc1	troponin C1, slow skeletal and cardiac type	NP_033419	-1.20	-2.38		●						
Gm10635	predicted gene 10635	NR_045336	-1.10	-2.42		●						
Mylk3	myosin light chain kinase 3	NM_175441	-1.04	-2.28		●				●		
Mecom	MDS1 and EVI1 complex locus	NM_007963	0.05	-1.76		●						
Rspo1	R-spondin 1	NP_619624	-0.80	-1.46		●						
Myl4	myosin, light polypeptide 4	NM_010858	-0.29	-1.36		●						(Polymenidou et al. [58])
Ddit4l	DNA-damage-inducible transcript 4-like	NM_030143	-0.65	-1.13		●						
Grm2	glutamate receptor, metabotropic 2	NM_001160353	-0.52	-1.12		●						
Cpne9	copine family member IX	NM_170673	-0.70	-1.04		●						
Ryr1	ryanodine receptor 1	NP_033135	-0.58	-0.98		●					● (up)	
Rassf3	Ras association domain family member 3	NP_620406	-0.26	-0.91		●						
Egr1	early growth response 1	NM_007913	-0.42	-0.90		●	(Chen-Plotkin et al. [17])				● (down)	
Kcnt2	potassium channel, subfamily T, member 2	NM_001081027	-0.54	-0.89		●				●		
Rims3	regulating synaptic membrane exocytosis 3	NM_182929	-0.38	-0.84		●		●				(Polymenidou et al. [58])
Pisd-ps3	phosphatidylserine decarboxylase, pseudogene 3	NR_003518	0.10	-0.86		●						
Kcnab3	potassium voltage-gated channel, shaker-related subfamily, beta member 3	NM_010599	-0.11	-0.86		●						(Polymenidou et al. [58])
Fos	FBJ osteosarcoma oncogene	NM_010234	-0.77	-0.84		●						
Scube1	signal peptide, CUB domain, EGF-like 1	NM_001271472	-0.43	-0.83		●					● (up)	
Kcnh5	potassium voltage-gated channel, subfamily H (eag-related), member 5	NM_172805	-0.45	-0.83		●						
Inf2	inverted formin, FH2 and WH2 domain containing	NM_198411	-0.35	-0.83		●						
Acvr1c	activin A receptor, type IC	NM_001111030	-0.13	-0.82		●						
Hipk4	homeodomain interacting protein kinase 4	NP_001028487	-0.51	-0.81		●						
Scrt1	scratch family zinc finger 1	NM_130893	-0.26	-0.77		●						
Hapln4	hyaluronan and proteoglycan link protein 4	NM_177900	-0.47	-0.75		●						(Polymenidou et al. [58])
Kif5a	kinesin family member 5A	NM_001039000	-0.52	-0.76		●						
Arhgap10	Rho GTPase activating protein 10	NP_001074833	0.00	-0.75		●						
Camk2n1	calcium/calmodulin-dependent protein kinase II inhibitor 1	NM_025451	-0.40	-0.74		●		●			● (down)	
Car10	carbonic anhydrase 10	NP_082572	-0.33	-0.73		●						
Camk4	calcium/calmodulin-dependent protein kinase IV	NM_009793	-0.24	-0.73		●				●		(Polymenidou et al. [58])
Rims1	regulating synaptic membrane exocytosis 1	NM_001012623	-0.42	-0.73		●	(Chen-Plotkin et al. [17])					(Polymenidou et al. [58])
Eipr1	EARP complex and GARP complex interacting protein 1	NM_201357	-0.68	-0.73		●						(Polymenidou et al. [58])
Rgs4	regulator of G-protein signaling 4	NM_009062	-0.52	-0.73		●	(Chen-Plotkin et al. [17])					
Rapgef4	Rap guanine nucleotide exchange factor (GEF) 4	NM_001204165	-0.23	-0.70		●					● (down)	
Dagla	diacylglycerol lipase, alpha	NM_198114	-0.43	-0.69		●						
Diras2	DIRAS family, GTP-binding RAS-like 2	NM_001024474	-0.52	-0.69		●	(Chen-Plotkin et al. [17])					
Gp1bb	glycoprotein Ib, beta polypeptide	NM_001001999	-0.65	-0.68		●						
Ano3	anoctamin 3	NM_001128103	-0.33	-0.67		●				●		
Grm3	glutamate receptor, metabotropic 3	NM_181850	-0.26	-0.67		●						
Nefm	neurofilament, medium polypeptide	NM_008691	-0.53	-0.66		●						(Polymenidou et al. [58])
Gabra3	gamma-aminobutyric acid (GABA) A receptor, subunit alpha 3	NM_008067	-0.57	-0.65		●						(Polymenidou et al. [58])
Rph3a	rabphilin 3A	NM_001302344	-0.39	-0.65		●	(Chen-Plotkin et al. [17])					
Tbr1	T-box brain gene 1	NM_009322	-0.51	-0.63		●						(Polymenidou et al. [58])
Satb1	special AT-rich sequence binding protein 1	NM_001163630	-0.39	-0.64		●						
Sh3gl2	SH3-domain GRB2-like 2	NM_019535	-0.22	-0.63		●	(Chen-Plotkin et al. [17])					
Ephb6	Eph receptor B6	NM_001146351	-0.37	-0.63		●	(Chen-Plotkin et al. [17])					(Polymenidou et al. [58])
Kcnh3	potassium voltage-gated channel, subfamily H (eag-related), member 3	NM_010601	-0.43	-0.62		●						
Nrgn	neurogranin	NM_022029	-0.35	-0.62		●	(Chen-Plotkin et al. [17])					
Cadm2	cell adhesion molecule 2	NM_001145977	-0.17	-0.61		●				●		
Camkk2	calcium/calmodulin-dependent protein kinase kinase 2, beta	NM_001199676	-0.44	-0.61		●	(Chen-Plotkin et al. [17])					
Rorb	RAR-related orphan receptor beta	NM_001043354	-0.34	-0.60		●						
Scn4b	sodium channel, type IV, beta	NP_001013408	0.05	-0.60		●		●				
Camk2a	calcium/calmodulin-dependent protein kinase II alpha	NM_001286809	-0.50	-0.60		●	(Chen-Plotkin et al. [17])					
Nrn1	neuritin 1	NM_153529	-0.24	-0.60		●	(Chen-Plotkin et al. [17])					
Cdk5r2	cyclin-dependent kinase 5, regulatory subunit 2 (p39)	NM_009872	-0.42	-0.60		●						
Rbfox3	RNA binding protein, fox-1 homolog (C. elegans) 3	NM_001024931	-0.34	-0.60		●						
Gca	grancalcin	NP_663498	0.08	-0.59		●						
Osbpl1a	oxysterol binding protein-like 1A	NM_001252489	-0.39	-0.59		●				●		
Vsnl1	visinin-like 1	NM_012038	-0.32	-0.59		●	(Chen-Plotkin et al. [17])			●		
Adcy1	adenylate cyclase 1	NM_009622	-0.25	-0.59		●	(Chen-Plotkin et al. [17])					(Polymenidou et al. [58])
Neurod6	neurogenic differentiation 6	NM_009717	-0.41	-0.59		●						(Polymenidou et al. [58])
AI593442	expressed sequence AI593442	NM_001286641	-0.19	-0.58		●						
Stx1a	syntaxin 1A (brain)	NM_016801	-0.28	-0.57		●	(Chen-Plotkin et al. [17])					(Polymenidou et al. [58])
Nat8l	N-acetyltransferase 8-like	NP_001001985	-0.34	-0.57		●						
3110035E14Rik	RIKEN cDNA 3110035E14 gene	NM_178399	-0.28	-0.57		●						(Polymenidou et al. [58])
Zbtb18	zinc finger and BTB domain containing 18	NM_001012330	-0.22	-0.57		●						
Hlf	hepatic leukemia factor	NM_172563	-0.39	-0.56		●						
Lynx1	Ly6/neurotoxin 1	NM_011838	-0.28	-0.56		●						(Polymenidou et al. [58])
Cadps2	Ca2+-dependent activator protein for secretion 2	NM_001252105	-0.53	-0.56		●						
Psd	pleckstrin and Sec7 domain containing	NM_133694	-0.47	-0.56		●						
Dhcr24	24-dehydrocholesterol reductase	NM_053272	-0.49	-0.54		●		●				
2900011O08Rik	RIKEN cDNA 2900011O08 gene	NM_144518	-0.53	-0.53		●						
Dlgap3	discs, large (Drosophila) homolog-associated protein 3	NM_001302081	-0.43	-0.52		●						(Polymenidou et al. [58])
Bhlhe40	basic helix-loop-helix family, member e40	NM_011498	-0.35	-0.53		●						
Pcp4	Purkinje cell protein 4	NM_008791	-0.04	-0.52		●	(Chen-Plotkin et al. [17])	●				
Cntnap1	contactin associated protein-like 1	NM_016782	-0.38	-0.52		●	(Chen-Plotkin et al. [17])					
Cep170b	centrosomal protein 170B	NM_001024602	-0.42	-0.51		●						
Plcxd2	phosphatidylinositol-specific phospholipase C, X domain containing 2	NM_001134480	-0.16	-0.50		●						
Cplx2	complexin 2	NM_009946	-0.26	-0.50		●						
Phyhip	phytanoyl-CoA hydroxylase interacting protein	NM_145981	-0.38	-0.50		●	(Chen-Plotkin et al. [17])					
Snap25	synaptosomal-associated protein 25	NM_001291056	-0.43	-0.50		●	(Chen-Plotkin et al. [17])					
Slc8a2	solute carrier family 8 (sodium/calcium exchanger), member 2	NM_001347561	-0.42	-0.50		●	(Chen-Plotkin et al. [17])					
D430041D05Rik	RIKEN cDNA D430041D05 gene	NM_001033347	-0.47	-0.49		●						
Tubb4a	tubulin, beta 4A class IVA	NM_009451	-0.38	-0.49		●						
Ppp3r1	protein phosphatase 3, regulatory subunit B, alpha isoform (calcineurin B, type I)	NM_024459	-0.25	-0.48		●	(Chen-Plotkin et al. [17])			●		
Epdr1	ependymin related protein 1 (zebrafish)	NM_134065	-0.35	-0.48		●		●		●		
Scn1b	sodium channel, voltage-gated, type I, beta	NM_011322	-0.20	-0.47		●						
Jph3	junctophilin 3	NM_020605	-0.39	-0.47		●		●				
Syt13	synaptotagmin XIII	NM_030725	-0.33	-0.45		●		●				(Polymenidou et al. [58])
Snap91	synaptosomal-associated protein 91	NM_001277982	-0.53	-0.45		●	(Chen-Plotkin et al. [17])					
Zfp365	zinc finger protein 365	NM_178679	-0.24	-0.44		●						
Atp1a1	ATPase, Na+/K+ transporting, alpha 1 polypeptide	NM_144900	-0.33	-0.44		●						

The down-regulated genes are indicated by the "-" sign in the columns of Log2 (TDP-43 cKO/Ctrl). The genes that have been reported to have altered mRNA levels in the FTLD patients and in the striatum or hippocampus upon TDP-43 depletion are indicated in the far right column

#### Up/down-regulated TDP-43 cKO mouse neocortex genes associated with inflammation, autophagy, and synaptic function

Significantly, 26 genes and 10 genes were up-regulated by > 2 fold in 3- and 12-month-old TDP-43 cKO mouse neocortex, respectively (Fig. 5a-(ii) and Table 1)**,** and most of them encoded the inflammatory proteins. Three out of these up-regulated genes, i.e. *Gfap*, *Serpina3a*, and *C4a/C4b*, were constitutively up-regulated at both the ages of 3 months and 12 months (Additional file 1: Figure S5a), as also quantified by qRT-PCR (Additional file 1: Figure S5b). Of the three genes, increase of *Gfap* and *Serpina3n* mRNAs in reactive astrocyte was reported in brain injury and in several neurodegeneration diseases [26, 93]. We also calculated and compared the intron sizes of TDP-43-regulated genes in the Ctrl and TDP-43 cKO mouse neocortex. It was found that the total lengths of the introns of down-regulated neocortex genes in TDP-43 cKO mice (the median values being 30,061 bp and 28,604 bp at the ages of 3 months and 12 months, respectively) were larger than those of control (the median value being 11,754 bp), whereas the trend was not observed for the up-regulated neocotex genes (the median values being 9968 bp and 13,098 bp at the ages of 3 months and 12 months, respectively) (Additional file 1: Figure S5c). To assess the empirical *P* values, we calculated the median values of the total intron lengths of 100 genes randomly selected from the annotated mouse protein-coding genes and the process was repeated for 10,000 times. Indeed, the down-regulated neocortex genes of the TDP-43 cKO mice at either the age of 3 months or 12 months possessed significantly longer total introns than expected, with the P values < 0.001. This result is consistent with the previous observation that down-regulated genes in striatum upon TDP-43 reduction tend to have long introns [58].

Increase of Gfap protein in the cortex and hippocampus of TDP-43 cKO mice at the ages of 3 months and 12 months was confirmed by Western blotting, respectively (Fig. 1b). Note that one additional band in GFAP immunoblotting in protein extracts of 3-month-old mice could be the isoform of GFAP protein. On the other hand, most of 20 down-regulated genes in the neocortex of 3-month-old TDP-43 cKO mice **(**Fig. 5a**)** encoded proteins involved in the functions of synapse, e.g. *Dlg3*, endosome, e.g. *Lamp5*, and autophagosome, e.g. *Tecpr1* **(**Table 1**)**. Furthermore, eight of these 20 down-regulated genes were constitutively repressed in the neocortex of TDP-43 cKO mice at the age of 12 month **(**Fig. 5a and Table 1**)**.

We also analyzed the altered expression of several genes by Western blotting. Firstly, autophagy defect was reported in *Tecprl* gene knockout mice with increased expression of an autophagy substrate, p62 [14]. As shown in Additional file 1: Figure S5d, the level of p62 protein was increased in the cortex of TDP-43 cKO mice at all ages analyzed. Secondly, consistent with the RNA-seq data **(**Additional file 1: Fig. S5a**)** and RT-qPCR analysis (Additional file 1: Figure S5b), the levels of SAP102 protein, which was encoded by the *Dlg3* gene mentioned above and involved in synaptic plasticity by regulating the recycling of NMDA receptor NMDAR [13], in the cortex and synaptosome of TDP-43 cKO mice were reduced in an age-dependent manner **(**Additional file 1: Figure S5e**)**. Since NMDA receptor (NMDAR)-mediated responses regulated the levels and activities of CaMKII family members [48], we also examined the levels of different synaptic proteins including the CaM kinase proteins CaMK4, NMDAR submit NR2b, and phospho-Erk1/2. Indeed, the amounts of these proteins were all greatly reduced in the cortex and/or synaptosome of 12-month-old TDP-43 cKO mice in comparison to the Ctrl mice **(**Additional file 1: Figure S5e**)**, while the amount of SAP102 was decreased in the cortex and synaptosome of early stage TDP-43 cKO mice. Thus, depletion of TDP-43 in the cortex indeed would down-regulate the expression of a specific set of genes and this could contribute in part to the impairment of synaptic functions (Additional file 1: Figure S4) and behaviour deficits (Fig. 2) in an age-dependent manner.

#### Mis-regulation of RNA processing

Abrrant RNA processing was increasingly recognized as a potential contributor to the development/ pathogenesis of neurological diseases [68]. Among the different RNA processing events, alternative splicing (AS) is one major mechanism for the enhancement of transcriptome diversity. A growing number of human diseases were correlated with RNA mis-splicing [3]. There are several different types of alternative splicing events including inclusion/exclusion of conserved (consitutively present in wild type RNAs) or non-conserved (cryptic)(absent in the wild type RNAs) exons as well as the alternative splicing site selection leading to extension of conserved exons. To investigate the regulatory role of AS in the forebrain neurons of TDP-43 cKO mice, we used the Cufflink [77] and MISO (Mixture of Isoforms) [40] programs to examine usage changes of alternatively spliced exons (ASEs) and poly(A) sites. In comparison to the Ctrl mice**,** 55 and 57 transcript processing events exhibited remarkably usage changes of ASEs or polyA sites in the neocortex of 3- and 12-month-old TDP-43 cKO mice, respectively, (Fig. 5b, Additional file 1: Figure S6 and S7). Most (> 85%) of these changed transcript events were ASEs **(**Fig. 5b and Table 2). Inclusion of cryptic exons was also observed (Additional file 1: Figure S6d and Table 2). Notably, some of these transcript events with siginificant usage changes were found only in 3-month-old TDP-43 cKO mice, e.g. *Pdp1*, or only in 12-month-old TDP-43 cKO mice, e.g. *Ranbp17*, while others were found at both ages **(**Table 2). For example, polyA site usage of the *Kctd2* transcript was altered in cKO mice at both ages and the *Polr1b* transcript was altered only in 3-month-old TDP-43 cKO mice (Additional file 1: Figure S7 and Table 2).

**Table 2:** Genes with altered mRNA processing patterns in the neocortex of TDP-43 cKO mice

	Gene	Location	Strand	Significant		Δmisoψ		White et al. [86]		References
				3 Months	12 Months	3 Months	12 Months	5 Months	20 Months	
Cryptic	Cdh22	Ch2:165183239-165183371	-	●	●	0.61	0.54			Jeong et al. [37]
Cryptic	Camk1g	Ch1:193368867-193368952	-	●	●	0.46	0.59			Jeong et al. [37]
Cryptic	Slc45a1	Ch4:150630400-150630454	-	●	●	0.32	0.13			Jeong et al. [37]
Cryptic	Synj2bp	Ch12:81509828-81510051	-	●	●	0.39	0.30			Jeong et al. [37]
Cryptic	Hgsnat	Ch8:25945949-25945996	-	●	●	0.22	0.19			Jeong et al. [37]
Cryptic	Adnp2	Ch18:80138153-80138304	-	●	●	0.45	0.49			Jeong et al. [37]
Cryptic	Abca8b	Ch11:109975240-109975477	-	●	●	0.18	0.18			
Cryptic	Upf3a	Ch8:13789928-13789967	+	●		0.17	0.07			
Cryptic	Letm1	Ch5:33779574-33779604	-	●		0.13	0.04			Jeong et al. [37]
Inclusion	Sort1	Ch3:108355472-108355570	+	●	●	0.35	0.40	●(exclusion)	●(exclusion)	Polymenidou et al. [58]
Inclusion	Islr2-02	Ch9:58200272-58200461	-	●	●	0.36	0.37			
Inclusion	Islr2-01	Ch9:58200272-58200443	-	●	●	0.30	0.29			
Inclusion	Bsg	Ch10:80136663-80136743	-	●	●	0.25	0.13			
Inclusion	Vps13d	Ch4:145099352-145099463	-	●	●	0.23	0.18		●(inclusion)	
Inclusion	Smg5	Ch3:88340649-88340763	+	●	●	0.20	0.10			
Inclusion	Smarca4	Ch9:21677953-21678051	+	●	●	0.20	0.24			
Inclusion	Uggt2	Ch14:119043908-119044028	-	●	●	0.19	0.08			
Inclusion	Elac2	Ch11:65005454-65005505	-	●	●	0.15	0.12			
Inclusion	Kcnmb4	Ch10:116443772-116443912	-	●	●	0.11	0.08			
Inclusion	Dnajc5	Ch2:181548926-181549000	+	●	●	0.10	0.13			Polymenidou et al. [58]
Inclusion	Sun1	Ch5:139230773-139230838	+	●	●	-0.14	0.32			
Inclusion	Pdp1-01	Ch4:11965614-11965648	-	●		0.40	0.24			
Inclusion	Tmem2	Ch19:21780171-21780252	+	●		0.40	0.19			
Inclusion	Zfp30	Ch7:29788049-29788175	+	●		0.32	-0.04			
Inclusion	Zkscan16	Ch4:58943943-58944160	+	●		0.29	0.18			
Inclusion	Nfia-02	Ch4:98081725-98081816	+	●		0.12	0.02			
Inclusion	Lrrk2	Ch15:91785371-91785527	+	●		0.06	-0.01			
Inclusion	Atxn1	Ch13:45849519-45849588	-	●		0.10	0.09			Polymenidou et al. [58]
Inclusion	Ranbp17	Ch11:33283908-33283990	-		●	-0.17	0.28			
Inclusion	Srr	Ch11:74919437-74919662	-		●	0.11	0.22			
Inclusion	Atad2b	Ch12:4970406-4970468	+		●	-0.03	0.15			
Inclusion	Kctd10	Ch5:114376771-114376866	-		●	0.05	0.06			
Inclusion	Pdp1-02	Ch4:11965614-11965648	-		●	#REF!	0.05			
Inclusion	Mettl22	Ch16:8482127-8482167	+		●	0.00	0.03			
Exclusion	Cobl	Ch11:12306958-12307128	-	●	●	-0.34	-0.41	●(inclusion)		
Exclusion	Scamp1	Ch13:94210577-94210678	-	●	●	-0.31	-0.26			
Exclusion	Ddx50	Ch10:62627521-62627682	-	●	●	-0.27	-0.37			
Exclusion	Kcnip2-02	Ch19:45797091-45797186	-	●	●	-0.23	-0.29			
Exclusion	Dtwd1	Ch2:126158410-126158553	+	●	●	-0.27	-0.19			
Exclusion	Nlgn3	ChX:101307075-101307134	+	●	●	-0.22	-0.15			
Exclusion	Lzts1	Ch8:69182213-69182331	-	●	●	-0.12	-0.15			
Exclusion	Nrxn1	Ch17:90701988-90702011	-	●	●	-0.09	-0.16			
Exclusion	Shisa4	Ch1:135373152-135373285	-	●	●	-0.07	-0.14		●(inclusion)	
Exclusion	Rdh13	Ch7:4444978-4445122	-	●	●	0.08	-0.26			
Exclusion	Atp11b	Ch3:35843571-35843696	+	●		-0.44	-0.28		●(inclusion)	
Exclusion	Gpatch1	Ch7:35281332-35281480	-	●		-0.36	0.00			
Exclusion	Kcnip2-01	Ch19:45796279-45796332	-	●		-0.21	-0.18			
Exclusion	Nfia-01	Ch4:98041551-98041679	+	●		-0.15	0.00			
Exclusion	Dzip3	Ch16:48951543-48952160	-	●		-0.13	0.01			
Exclusion	Cacna1b-01	Ch2:24618255-24618362	-	●		-0.04	0.01			
Exclusion	Tmcc2	Ch1:132380657-132381172	-	●		-0.05	0.01			
Exclusion	Pcm1-01	Ch8:41313302-41313460	+	●		-0.08	-0.01			
Exclusion	Cdk19-02	Ch10:40466638-40466769	+		●	-0.02	-0.04			
Exclusion	Max	Ch12:76939430-76939514	-		●	-0.08	-0.05			
Exclusion	Phactr2	Ch10:13253342-13253879	-		●	-0.16	-0.08			
Exclusion	Cdk19-01	Ch10:40454015-40454072	+		●	0.02	-0.05			
Exclusion	Rimbp2	Ch5:128846922-128846991	-		●	-0.01	-0.09			
Exclusion	Clasp1-01	Ch1:118512175-118512222	+		●	0.02	-0.12			
Exclusion	Clasp1-03	Ch1:118541675-118541698	+		●	0.07	-0.12			
Exclusion	Cntln-01	Ch4:84984369-84984500	+		●	-0.02	-0.20			
Exclusion	Agfg1	Ch1:82891460-82891507	+		●	-0.16	-0.26			
Exclusion	Repin1	Ch6:48594862-48594976	+		●	-0.07	-0.24			
Exclusion	Ppp3ca	Ch3:136932011-136932040	+		●	-0.09	-0.11	●(inclusion)	●(inclusion)	
Exclusion	Hspa13	Ch16:75758632-75758727	-		●	0.01	-0.08			
Extension	Wbscr22	Ch5:135063781-135063921	-	●	●	0.17	0.19			Jeong et al. [37]
Extension	Chga	Ch12:102558298-102558559	+	●	●	0.08	0.06			Jeong et al. [37]
Extension	Bptf	Ch11:107054456-107055318	-	●	●	-0.10	-0.11			
PolyA extension	Rapgefl1	Ch11:98851076-98857648	-	●	●	0.17	0.21			Jeong et al. [37]
PolyA extension	Kctd2	Ch11:115430313-115433591	+	●	●	0.14	0.11			
PolyA extension	Kcnj4	Ch15:79505196-79505875	-	●	●	0.09	0.06			
PolyA extension	Ergic1	Ch17:26655067-26658770	+	●	●	0.05	0.09			
PolyA extension	Syt17	Ch7:118378587-118379874	-	●	●	0.06	0.16			
PolyA extension	Polr1b	Ch2: 129125214:129126791	+	●		0.14	0.13			
PolyA extension	Elk1	ChX:20932683-20935548	-		●	0.19	0.22			
PolyA extension	Ppp3cc	Ch14:70214901-70215786	-		●	0.08	0.09			

The ψ (PSI, percentage of spliced in) score was defined as the percentage of transcripts containing the alternative splicing events and/ or alternative poly(A) site usage. The mRNAs with increase of splcing events, i.e. conserved exon inclusion/ exclusion, cryptic exon inclusion, and exon extension, as well as change of poly(A) site usage are indicated by Δ ψ > 0, mRNA with decrease of the processing events are indicated by Δ ψ < 0. Unpaired t test was used to calculate the significance from data of 3 independent samples. Note that changes of the pre-mRNA processing events of several genes including *Cob1* in the TDP-43 cKO mice are oppisite to those observed in the TDP-43(Q331K) knock-in mice (White et al. [86])

We then examined some of the differential RNA processing events by RT-qPCR and/or semi-quantitative RT-PCR (Additional file 1: Figure S8 and Figure S9). For instance, Sortilin 1 (*Sort1*), a member of a family of cellular vacuolar protein sorting 10 (VSP10)-domain receptors, was primarily expressed in neurons and a key player in regulating the neuronal viability and function [87]. It was proposed that TDP-43 regulates the splicing of *Sort1* mRNA in mouse striatum and cell lines [58, 60]. We confirmed *Sort1*(wt) as the main mRNA isoform encoding sortilin in mouse cortex by RT-PCR and Western blotting (Additional file 1: Figure S9a and b). However, depletion of TDP-43 expression in the neocortex of TDP-43 cKO mice led to the accumulation at all stages of the higher molecular weight RNA isoform *Sort1*(e17b) encoding a non-functional progranulin receptor Sortilin 1(e17b) [60] (Additional file 1: Figure S8a, S9a and b). Quantification analysis of *Sort1* mRNA levels by qRT-PCR in different mouse brain areas confirmed this observation in both 3- and 12-month-old TDP-43 cKO mice (Additional file 1: Figure S8a). Other events of conserved and cryptic exon inclusions induced by depletion of TDP-43 were also comfirmed by qRT-PCR, as exemplified for *Dnajc5*,*CaMK1g,*and *Adnp2*, respectively (Additional file 1: Figure S8). Notably, the expression levels of the wild type isoforms of *Sort 1* and *Dnajc5* in the cortex of TDP-43 cKO mice were unaltered (Additional file 1: Figure S9c), while those of *CaMK1g* and *Adnp2* were unchanged in the cortex of 3-month-old TDP-43 cKO mice (upper panels, Additional file 1: Figure S9d) but moderately increased at the age of 12 months (lower panels, Additional file 1: Figure S9d).

#### Mis-regulation of circular RNA processing in TDP-43 cKO mice

**Fig. 6:**
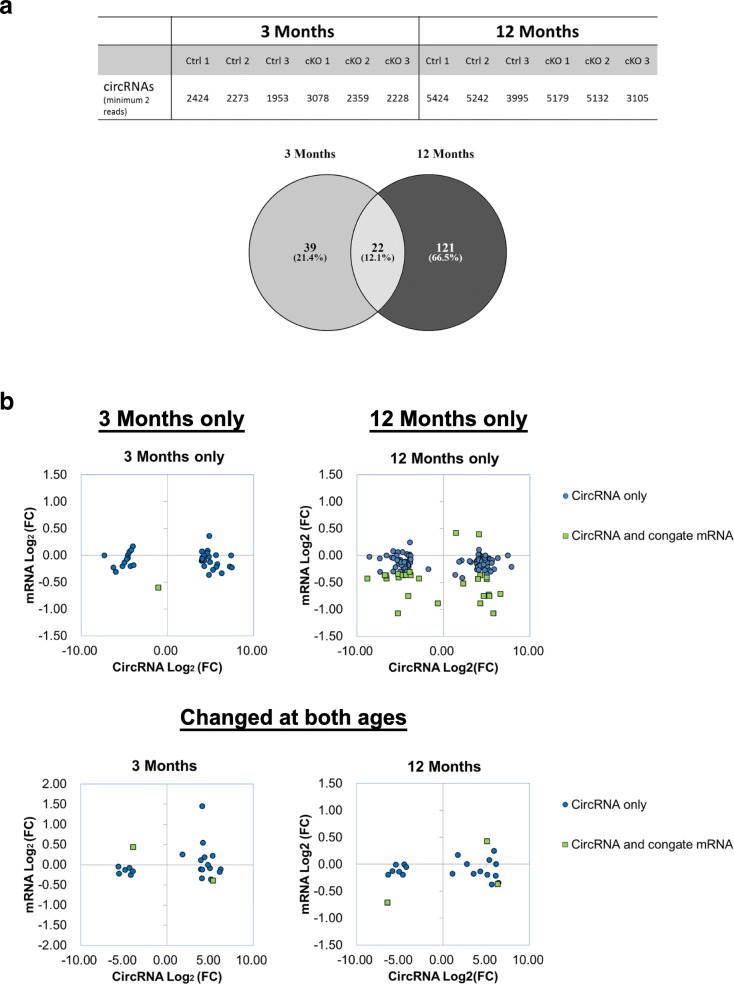
RNA-Seq analysis of circRNAs. **a** Upper panel, list of the numbers of cirRNAs in the neocortex of 3 each of TDP-43 cKO and Ctrl mice at the ages of 3 months and 12 months, respectively. Lower plot, venn diagram showing the numbers of cortex circRNAs the expression levels of which were different between the TDP-43 cKO and Ctrl mice at the ages of 3 months and 12 months, respectively. Note that the levels of 22 cricRNAs were changed at both ages. **b** Upper panels, scatter plot showing the correlation of log_2_ fold change (FC, TDP-43 cKO/Ctrl) of cortex circRNAs (x axis) and their cognate linear mRNAs (y axis) the levels of which were altered in TDP-43 cKO mice only at the age of 3 months or only at the age of 12 months in comparison to Ctrls. Lower panels, scatter plot showing the correlation of log_2_ FC of neocortex circRNAs (x axis) and their cognate linear RNAs (y axis) the levels of which were changed at both the ages of 3 months and 12 months in TDP-43 cKO mice in comparison to Ctrls. Blue circles indicate changes of the expression levels of only the circRNAs, while green squares indicate changes of the expression levels of both the circRNAs and their cognate mRNAs counterparts

Circular RNAs (circRNAs) are RNA molecules in which a covalent linkage termed a “backsplice” has formed between a downstream 3′ splice site and an upstream 5′ splice site in a linear pre-messenger RNA [73]. Previous studies have lead to the identification of thousands of circRNAs in diverse species [15, 31, 36, 67, 84] that are enriched in neuronal tissues and may play specific roles in neuronal processes [29, 65, 92]. Analysis of our RNA-seq data by the NCLscan pipeline [19] revealed that the expression levels of 182 circRNAs in the neocortex were significantly different between the TDP-43 cKO and Ctrl mice (Fig. 6 and Additional file 2: Table S1). Among them, the expression levels of 22 circRNAs were significantly altered in the neocortex of 3- as well as 12-month-old TDP-43 cKO mice when compared to the Ctrl mice. The levels of 39 circRNAs were changed only at the age of 3 months and 121 circRNAs were altered only at the age of 12 months (Fig. 6a and Additional file 2: Table S1). Notably, a considerable percentage of the circRNAs and their corresponding co-linear mRNA isoforms exhibited different changes of their expression levels in TDK-43 cKO in comparison to the Ctrl mice (Fig. 6b). This result reflects the previous observation that circRNAs and their co-linear counterparts could compete with each other for biogenesis during splicing [6, 16]. The biological significance of the alterations of expression levels of the circRNAs in the TDP-43 cKO mouse neocortex await to be examined.

## Discussion

TDP-43 proteinopathy is assocaited with more than 95% of ALS (ALS-TDP) and more than 50% of FTLD (FTLD-TDP) [47]. A gain-of-toxicity mechanism for early pathogenesis of FTLD-TDP or ALS-TDP has been suggested in view of the aberrant RNA metabolism and/or purturbed autoregulation of TDP-43 caused by mutant TDP-43 in different mouse models [5, 24, 58, 59, 86, 89]. One the other hand, a common characteristic of TDP-43 pathology at the later stage of FTLD-TDP or ALS-TDP is the loss of nuclear TDP-43 with concomitant cytoplasmic TDP-43 accumulation in neurons and glia [54]. This nuclear clearing provides a disease mechanism that is at least partially driven by the loss of normal TDP-43 function in the nucleus, as supported by studies of different mouse models with knockout or knockdown of TDP-43 expression [89, 91]. The presence of the cytoplasmic TDP-43(+) inclusions would also cause gain of one or more cytotoxic properties [44].

**Fig. 7:**
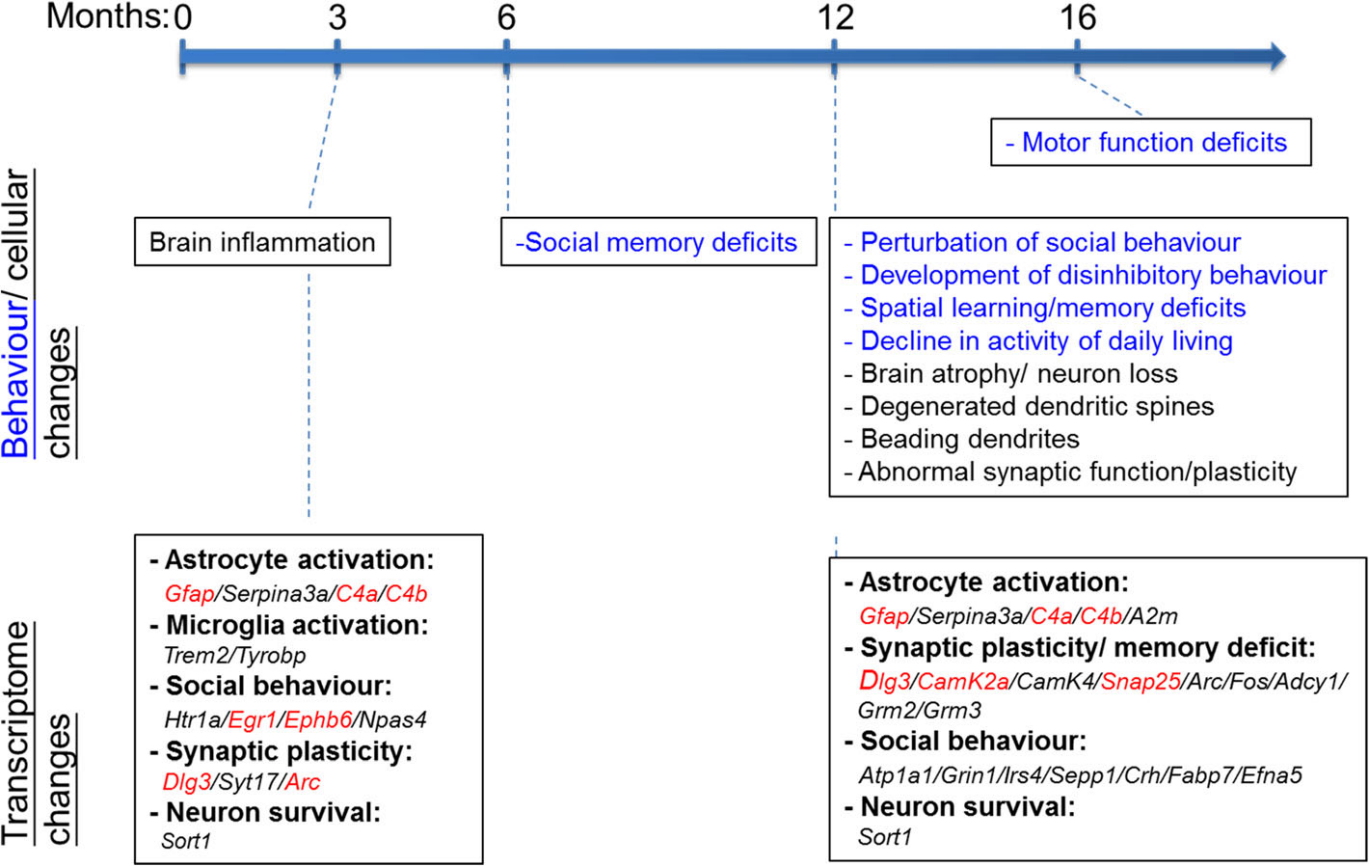
Age-dependence of FTLD-like pathogenesis of TDP-43 cKO mice. The ages (months) of the detection of different behaviour (blue), cellular, and molecular pathologies in TDP-43 cKO mice upon conditional depletion of TDP-43 in their forebrain neurons are indicated along the time line on the top. Also listed in the 2 lower boxes are mis-regulated genes associated with specific pathogenic event, e.g. *Gfap*/ *Serpina3a*/ *C4a*/ *C4b*/ *A2m* and astrocyte activation, etc., are identified by RNA-Seq analysis of the TDP-43 cKO mouse neocortex on 3 and 12 months (genes altered in FTLD patients are indicated by red color), respectively. For more details, see text

As summerized in Fig. 7, this study shows that CaMKII-directed conditional depletion of TDP-43 expression in the forebrain neurons has adverse effects on the mice, leading to shorter life span and a range of age-dependent phenotypes on the behavioural, cellular, as well as molecular levels that mimic FTLD, especially bvFTLD [61, 75]. Specifically, depletion of TDP-43 in *αCaMKII*-expressing neurons in the mouse forebrain (Fig. 1) results in progressive perturbation of social behaviour (Fig. 2a), development of dementia-like behaviour (Fig. 2b), and impairment of learning/memory (Fig. 2c). The behaviour deficits are accompanied with brain atrophy and neurodegeneration in the cortical hippocampal region and massive astrocytosis (Fig. 3-4). These findings together with analysis of the transcriptomes/gene expression profiles of mouse neocortex at the pre-symptomatic and post-symptomatic strages (Fig. 5, Fig. 6 and Additional file 1: Figures S5-S10) demonstrate the function of TDP-43 in cognition, and synaptic function in the adult brain. Notably, the large neurons in cortical layer V of TDP-43 cKO mice are more voulunerable to TDP-43 depletion (Fig. 4). This data is consisitent with the finding by Yang et al. [91], in which 10% reduction of TDP-43 protein level in the forebrain region of a TDP-43 knockdown mouse model could causes ~ 25% loss of large neurons in cortical layer V. Thus, there appears to be a selective vulnerability of the forebrain neurons, with distinct neuronal populations in different cortical layers compromised by the depletion of TDP-43. Notably, depletion of TDP-43 did not affect the sensory neurons of above mouse model [91]. Overall, this study demonstrates that loss-of-function of TDP-43 in the forebrain neurons would lead to a range of pathological changes of the mice on the phenotypic, molecular, and cellular levels that mimic those in FTLD-TDP.

Since one-fifth of the genes the expression of which are altered in TDP-43 cKO mice are also affected in FTLD patients [17] (Table 1), mis-regulation of TDP-43 RNA targets through the loss of TDP-43 function could at least in part contribute to the impairment of synaptic functions and disease pathogenesis of FTLD-TDP.

Comparison of our RNA-seq data (Table 1) to the Mouse Genome Informatics database (MGI) [8] has revealed that the mRNA levels of genes associated with anxiety-related behaviour and/ or social behaviours are significantly altered in the neocortex of TDP-43 cKO mice (Additional file 3: Table S2)**.** Notably, *Slc6a3*, a gene encoding a sodium-dependent dopamine transporter and associated with anxiety disorder in autism spectrum disorder [56], is upregulated (by 4 fold) in the neocortex of TDP-43 cKO mice but only at the age of 3 months (Additional file 3: Table S2) [7, 9, 23, 28, 32, 39, 41, 50, 51, 66, 69, 90]. On the other hand, 16 of the rest 17 anxiety- and/or social behaviour–related genes listed in Additional file 3: Table S2 are up- or down-regulated in the neocortex TDP-43 cKO mice mainly at the age of 12 months when the behaviour abnormality shows up (Fig. 2). Egr1 was also demostrated in a TDP-43^Q331K^ knockin mice at the age of 20 months (Table 1) [86].

Similarly, the cognition deficencies of the TDP-43 cKO mice are also associated with altered expression levels of specific genes, particularly those involved in synaptic transmission, as revealed by the transcriptome anaysis (Table 1 and Additional file 1: Figure S10) and electro-physiology measurement (Additional file 1: Figure S4). Besides those illustrated in Additional file 1: Figure S5, the expression level of *dlg3*, which encodes a major excitatory postsynaptic density protein SAP102 important in NMDARs recycling [95], is significantly down-regulated in 3-month-old TDP-43 cKO mice as compared with Ctrl mice (Table 1). In addition, several other synaptic function-associated genes, e.g. *dlgap3, snap25,* are down-regulated in the cortex of 12-month-old TDP-43 cKO mice (Table 1). Among the proteins encoded by these genes, Dlgap3 (PSD95-associated protein 3) is an excitatory postsynaptic protein implicated in the pathogenesis of obsessive-compulsive behaviours [85], and Snap-25 is a component of the SNARE protein complex and a promising cerebrospinal fluid biomarker for synapse degeneration in Alzheimer’s disease [10]. With respect to the abnormal LTP and LTD (Additional file 1: Figure S4), the protein levels of CaMKII and p-Erk (Additional file 1: Figure S5c and d) are decreased in the cortex of TDP-43 cKO mice. Altogether, it appears that combined deficiencies of the expression of a set of forebrain genes contributed to the age-dependent cognition impairment of TDP-43 cKO mice. However, down-regulation of synaptic genes could also be the results from the degeneration of the according brain region with neuron loss in TDP-43 cKO mice.

Patients with dementia (e.g., FTLD) often exhibit activation of inflammatory reponse [27]. In TDP-43 cKO mice, the progressive increase of astrocytosis in SLM region of hippocampus and RS region of the cortex (Additional file 1: Figure S3) is associated with upregulation of a range of inflammatory genes, including *Gfap*, *C4a/C4b*, *Serpina3n*, and *A2m,* at both 3- and 12 months of age (Fig. 7 and Table 1). Particularly, *Serpina3n*, is a marker of persistent reactive gliosis response induced in inflammation [93] and in ALS [25]. Several other genes induced in microglia activation, e.g., *Cst7* and *Clec7a*, are also upregulated in TDP-43 cKO mice (Fig. 5d and Table 1). Taken together, the transcriptome analysis indicates that chronic neuroinflammation in TDP-43 cKO mice and by implication in FTLD-TDP patients’ results in part from mis-regulation of these genes.

Depletion of TDP-43 in the mouse forebrain also results in aberrant splicing of ~ 50 RNA transcripts in the cortex of 3- and/ or 12-month-old TDP-43 cKO mice (Fig. 5 and Table 2). Importantly, among these transcripts, *Sort1*, *Adnp2*, and *Cdh22*, the mutations or functional variants of which are associated with aging or neurodegenerative disorders [35, 42]. Furthermore, aberrant splcing of several genes including *Dnajc5, Sort1, Pdp1*, and *Kcnip2,* which also occus in TDP-43 knockdown cells of the striatum [58], does not affect the levels of their wild type mRNA isoforms (Additional file 1: Figure S9c). Consistently, the amounts of *Dnajc5* protein in the cortex of TDP-43 cKO and Ctrl mice are similar (data not shown). On the other hand, the truncated sortilin protein accumulates in the cortex of TDP-43 cKO mice at different ages (Additional file 1: Figure S9b). Interestingly, decreasing the *Sort1* e17b inclusion was reported by TDP-43^Q331K^ knock-in mice with a mild FTD phenotype [86](Table 1). Since sortilin is a major neuronal APOE receptor [12], the truncated *Sort1* (e17b) could act as a decoy receptor [60] competing with the wild type sortilin in the cortex of TDP-43 cKO mice thus affecting the neuronal viability and causing neurodegenertion.

Finally, cryptic exons are present in transcripts from a set of genes, including *Camk1g*, *Hgsnat*, *Synj2bp*, *Adnp2*, and *Abca8b,* in the neocortex of 3- and/or 12-month-old TDP-43 cKO mice (Table 2). Intriguingly, inclusion of the cryptic exon in *CaMK1g* would result in the loss-of-function of CaMK1γ that has been implicated in aging and ALS [22]. Besides *CaMK1g* and other gene transcripts reported previously [37, 46], we identified some novel cryptic exon inclusion events in several transcripts upon TDP-43 depletion (Table 2). Notably, cryptic exon inclusions often introduce premature termination codons (PTCs) and thereby result in nonsense-mediated decay (NMD) [34] of the inserted RNA transcripts. It has also been reported that TDP-43 could autoregulate itself through NMD [58]. Thus, we speculate that the accumulation of a portion of the cryptic exon-containing transcripts in the cortex of TDP-43 cKO mice might result from loss of TDP-43 function in NMD of these transcripts.

## Conclusions

In summary, we have generated a conditional mouse model (TDP-43 cKO) with depletion of TDP-43 in the neurons of cortex and hippocampus. The TDP-43 cKO mice exhibit a spectrum of age-dependent social behaviour change, dementia-like behaviour, impairment of the cognition functions, and decline of ADL. The development of neurodegenerative pathology in TDP-43 cKO mice is closely associated with their behaviour and cognition changes, both of which are well correlated with the age-dependent alterations of the cortex transcriptomes. Notably, the transcriptomopathies of the TDP-43 cKO mouse cortex consist of changes of mRNA levels as well as pre-mRNA splicing patterns of many genes. Related to the latter changes, alternative splicing is known to play an essential role in brain function and mutations in factors involved in splicing regulation cause a range of neurological diseases [68]. Transcriptome analyse of autopsied brains from patients with ALS/FTLD reported have identified thousands of alternative splicing changes in part regulated by hnRNP [59] including TDP-43.

Overall, this study not only supports that loss-of-function of TDP-43 could be a major cause for FTLD-TDP pathogenesis, but also suggests a list of potential TDP-43 target genes that may be useful for future therapeutic development of FTLD-TDP.

## Materials and methods

### Generation of TDP-43 conditional knockout mouse

The *Tardbp* allele was knocked out specifically in the postmitotic pyramidal neurons in the forebrain by crossing mice carrying the *Tardbp* conditional allele (*Tardbp*^lx^) with mice carrying a Cre-recombinase transgene driven by the *CaMKIIα*-promoter. The viability and weight of the mice were monitored regularly. Genotyping of the mice was performed by PCR of genomic DNAs from the tail biopsies.

### RNA-seq analysis

For RNA-seq, the rRNA-depleted cortical RNAs from three biological replicates of sex-matched TDP-43 cKO as well as littermate Ctrl mice were converted to cDNAs and sequenced them in a strand-specific manner at National Center of Genomic Medicine (NCGM).. The RNAs were extracted from intact mouse cortical tissues and their concentrations determined using NanoDrop 8000 (Thermo Scientific). The RNA integrity was determined by Fragment Analyzer (Advanced Analytical Technologies). cDNAs from 5 μg of total RNA was used as an input material for library preparation using TruSeq RNA Sample Preparation Kit v2 (Illumina). Insert sizes of the libraries were confirmed using Fragment Analyzer (Advanced Analytical Technologies). The libraries were multiplexed and then sequenced on Illumina HiSeq2000 (Illumina) to generate 50 M of pair end 100 base pair reads per library. Data were processed using the TopHat, Cufflinks [77] and MISO [40].

### RNA-seq normalization

The number of mapped fragments per kilobase of exon, per million mapped reads (FPKM) for each annotated protein-coding gene was determined to establish a metric of normalized gene expression. Approximately 80~85% of annotated protein-coding genes in mouse satisfied at least 1 FPKM in either condition. To judge the significance of differences between TDP-43 cKO and Ctrl mice, false discovery rates (q < 0.05) were used as the criteria for different replicates. The q value is an adjusted *P* value taking in to account the false discovery rate (FDR). We calculated the q value because the expression levels of thousands of genes from a small sample set (3 individual mice) were measured. The expression levels of transcripts in the neocortex of TDP-43 cKO mice relative to Ctrl mice were represented by log2 transformed values.

### Analysis of alternative splicng

The ψ (PSI, percentage of spliced in) score was defined as the percentage of transcripts containing the alternative splicing events and/ or alternative poly(A) site usage. The mRNAs with increase of splcing events, i.e. conserved exon inclusion/ exclusion, cryptic exon inclusion, and exon extension, as well as change of poly(A) site usage are indicated by Δ ψ > 0. mRNAs with decrease of the processing events are indicated by Δ ψ < 0. Multiple t test was used to calculate the significance from data of 3 independent samples and the Holm-Sidak method was used to correct for multiple t test.

### Clustering analysis of protein-coding and noncoding transcripts

For the clustering, transcripts with an estimated expression of a minimum of 0.2 FPKM in both TDP-43 cKO and Ctrl mice were selected for analysis. The list of protein-coding transcripts was compiled based on the RefSeq/Enterz/Vega definitions.

### Identification and analysis of circRNAs

circRNAs were identified by NCLscan (version 1.6; https://github.com/TreesLab/NCLscan/TreesLab/NCLscan) [20], which was reported to outperform other publicly-available tools in terms of precision and to be robust to background noise [19, 94], on the basis of the mouse reference genome (GRCm38) and the GENCODE annotation (version M10). The differential expression analysis was performed by DEseq2 [2] and edgeR [64], in which the circRNA supporting reads were normalized by Relative Log Expression (RLE) and Trimmed Mean of M-values (TMM), respectively. The *P* values were evaluated by the Wald test (DEseq2) and the Fisher’s exact test (edgeR), and then adjusted by the Benjamini-Hochberg procedure. In this study, the significantly differential expression of circRNAs between different stages should satisfy DEseq adjusted *P* < 0.05 and edgeR adjusted P < 0.05 simultaneously.

### Quantitative reverse transcription PCR (qRT-PCR)

Total RNAs were extracted from the cortex, hippocampus, and cerebellum of 3-and 12-month-old TDP-43 cKO mice and their Ctrl littermates (*N* = 6 for each genotype) using TRIzol reagents (Thermo Fisher Scientific). Synthesis of cDNA followed the manufacturer’s protocol (Invitrogen). qRT-PCR was performed using Roche qPCR FastMix (Roche). Primers were designed using a primer design software (LightCycler Probe Design Software 2.0 from Roche). The expression levels were normalized to *gapdh*, and data are represented as fold change relative to the Ctrl mRNA levels. Significant differences were determined using un-paired *t* tests.

### Nesting behaviour

Single-housed mice were transferred into a new cage with nest-building material, a 5 × 5 cm square of white compressed cotton pads (Nestlets TM; Ancare, Bellmore, NY) in a random corner. After 6, 24, and 48 h, nest building was scored on a scale of 0–5, as previously described [21]. All data are shown means ± SEM and analyzed using un-paired *t* tests.

### Social interaction test

This test has been successfully employed to study social affiliation and interest in social novelty or social discrimination (social memory). The test was performed as described previously [70]. Briefly, in the first 10-min session, a test mouse was placed in the center of the three-chamber unit, where two empty wire cages were located in the left and right chambers to habituate the test mouse. The mouse was allowed to freely explore each chamber. In the second 10-min session, an age- and gender-matched C57BL/6J mouse (S1) that had never been exposed to the test mouse, was placed in one of the two wire cages. The wire cage on the other side remained empty (E). Then, the test mouse was placed in the center, and allowed to freely explore the chamber for 10 min. The test mouse was removed and in the last 10-min session, a second age- and gender-matched C57BL/6J stranger mouse (S2) that had never been exposed to the test mouse, was placed in one wire cage, which previously served as an empty cage. Thus, the test mouse would now have the choice between a mouse that was already familiar (S1) and a new stranger mouse (S2). The test mouse was placed in the center, and allowed to freely explore the chamber for 10 min. The movement of the mouse was recorded by a camera. The recorded video file was further analyzed by off-line video tracking software (EthoVision XT 7.0). Time spent in each chamber, and time spent within a 5 cm radius proximal to each wire cage were measured. All data shown are means ± SEM and analyzed using two-way ANOVA with Bonferroni’s post hoc analysis.

### Light/ dark box test

The light–dark box was custom made (45 × 20 × 20 cm) and divided into two parts: 1/3 was painted black, covered by the lid, and separated from the white compartment by the wall containing an opening (13 × 5 cm) at the floor level. The light side was illuminated by two 40 W light bulbs 50 cm above the floor. Each mouse was released in the center of the light compartment (facing away from the opening) and allowed to explore the area for 10 min. Video tracking equipment and software (EthoVision XT 8, Noldus) were used for following the animal’s position and movement. In addition, rearings and attempts (stretched attend postures at the opening) to enter either the light or the dark compartment were recorded manually.

### Rotarod rod test

Mice were trained at the age of 2 months and then subjected to rotarod test monthly. The latencies before falling from the rod was recorded for 3 consecutive days.

### Immunohistochemistry and histochemmistry staining

Mice were anesthetized and perfused with 4% paraformaldehyde. The hemispheres were embedded in paraffin, sliced into 10-μm sections, and mounted on slices. For immunostaining, the sections were washed with 0.1 M PBS buffer, quenched by 1% H_2_O_2_, blocked in serum with 0.05% Triton X-100 for 30 min, and incubated in rabbit anti-TDP-43 (Genetex, 1:1000), mouse anti-glial fibrillary acidic protein (GFAP) (1:1000), mouse anti-SMI-32 (1:1000) and mouse anti-NeuN (Milipore, 1:200) at 4 °C overnight. After rinsing in PBS, the sections were incubated with biotinnlated goat antibody (Vector) for 60 min, followed by incubation with the avidin–biotin complex (Vector) for 45 min. The reaction products were developed by 3,3′-diaminobenzidine (Sigma, St Louis, MO).

To identify the cellular localization of TDP-43, slices were combined with hematoxylin. The hematoxylin and eosin (H&E) stain was used to visualize the overall morphology of the mouse brain. Nissl staining was applied to characterize the hippocampus size, cortical layer thickness, and neuron number. All data are presented as the mean with standard error, and statistical significance was tested by paired t-test.

### Electrophysiology

Standard protocol was followed as described previously, with some modifications. In short, the mice were decapitated and their brains rapidly removed and placed in ice-cold artificial CSF (ACSF). 450 μm thick sections of isolated mouse hippocampus were transferred into an interface-type holding chamber in oxygenated ACSF (95% O_2_ and 5% CO_2_) at room temperature to allow recovery for at least 90 min before the recording. The field excitatory postsynaptic potential (fEPSP) at Schaffer collateral branches in CA1 region of the hippocampal slices was recorded. Three trains of stimuli at 100 Hz separated by 60 s were applied for LTP induction, and low frequency stimulation (1 Hz for 15 min) were applied for LTD induction. The slopes of the fEPSP were measured and the synaptic responses were normalized to the average of the baseline. All data are presented as the mean with mean standard deviation. Statistical significance was tested by paired t-test.

### Statistical analyses

GraphPad Prism software was used for most statistical analysis. The statistical significance between means of control and TDP-43 cKO mouse groups was calculated by the two-tailed Student’s t-test. For comparisons involving more than two groups, one-way or two way ANOVA were used. Data are presented as measures for group means, and *P* < 0.05 was considered significant. Numbers of animals per group used in each experiment are indicated in figure legends.

## Additional files

Additional file 1: **Figure S1.** Altered activity of daily living (ADL) of TDP-43 cKO mice. **Figure S2.** Immunohistochemistry staining of brain slices. **Figure S3.** Persisting reactive astrocytosis in TDP-43 cKO mouse forebrain. **Figure S4.** Electrophysiology measurements. **Figure S5.** Mis-regulated genes in TDP-43 cKO mice. **Figure S6.** Alternations of the processing events of cortical RNAs in TDP-43 cKO mice. **Figure S7.** The alternative uses of poly(A) sites in the cortical RNAs of TDP-43 cKO mice. **Figure S8.** qRT-PCR validation of RNA splicing events altered in 3- and 12-month-old TDP-43 cKO mice. **Figure S9.** Validation of altered splicing events in TDP-43 cKO mice. **Figure S10.** Calcium signaling and synaptic long term potentiation pathway analysis using Ingenuity Pathway Analysis (IPA). (PDF 2990 kb)

Additional file 2: **Table S1.** List of the chromosome numbers, donor positions, acceptors positions and gene names of the transcripts from which the individual circularRNAs change in the neocortex of 3- and 12-month-old TDP-43 cKO mice, but not Ctrl mice are listed. (PDF 141 kb)

Additional file 3: **Table S2.** Changes of the mRNA levels of social behaviour-related genes in the neocortex of TDP-43 cKO mice relative to Ctrl mice. (PDF 72 kb)
