# Supplementary material for: Transcriptomopathies of pre- and post-symptomatic frontotemporal dementia-like mice with TDP-43 depletion in forebrain neurons
Source: Acta Neuropathol Commun. 2019 Mar 29;7:50. doi: 10.1186/s40478-019-0674-x (PMC6440020; doi:10.1186/s40478-019-0674-x)
Supplement: Supplementary file 1 — Figure S1. Altered activity of daily living (ADL) of TDP-43 cKO mice. Figure S2. Immunohistochemistry staining of brain slices. Figure S3. Persisting reactive astrocytosis in TDP-43 cKO mouse forebrain. Figure S4. Electrophysiology measurements. Figure S5. Mis-regulated genes in TDP-43 cKO mice. Figure S6. Alternations of the processing events of cortical RNAs in TDP-43 cKO mice. Figure S7. The alternative uses of poly(A) sites in the cortical RNAs of TDP-43 cKO mice. Figure S8. qRT-PCR validation of RNA splicing events altered in 3- and 12-month-old TDP-43 cKO mice. Figure S9. Validation of altered splicing events in TDP-43 cKO mice. Figure S10. Calcium signaling and synaptic long term potentiation pathway analysis using Ingenuity Pathway Analysis (IPA). (PDF 2990 kb) [file 40478_2019_674_MOESM1_ESM.pdf]

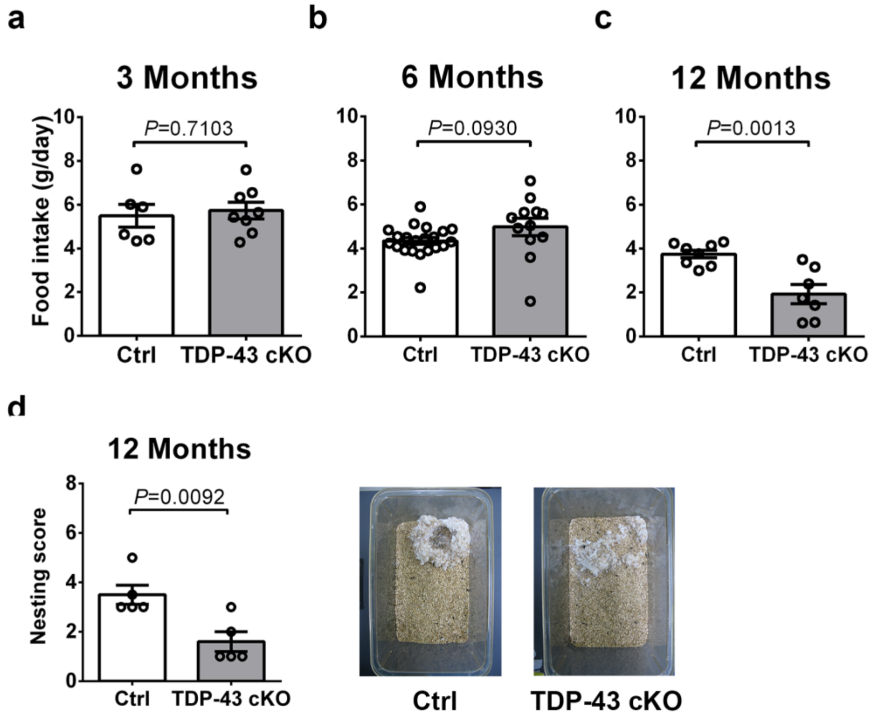

Supplemental Figure 1. Altered activity of daily living (ADL) of TDP-43 cKO mice. The daily food intake (grams per day) by Ctrl and TDP-43 cKO mice at the age of 3 months **(a)**, 6 months **(b)**, and 12 months **(c)** was measured and averaged over a period of 7 days. The mean (N > 7/group) values are compared in the histograms. Statistically analyzed in the histograms by unpaired t test with error bars reported as SEM, $P<0.05$ was considered significant. **d,** Comparison of the nest construction behaviour. Bar graph shows the results derived from the chip shaving nest construction experiments, in which TDP-43 cKO mice built nests of poorer quality in comparison to Ctrl mice. Represented images of the nests built are shown in the right panels. Statistical analysis was done by unpaired t test (N=5/group) with error bars reported as SEM, $P<0.05$ was considered significant.

Supplemental_Fig_S2

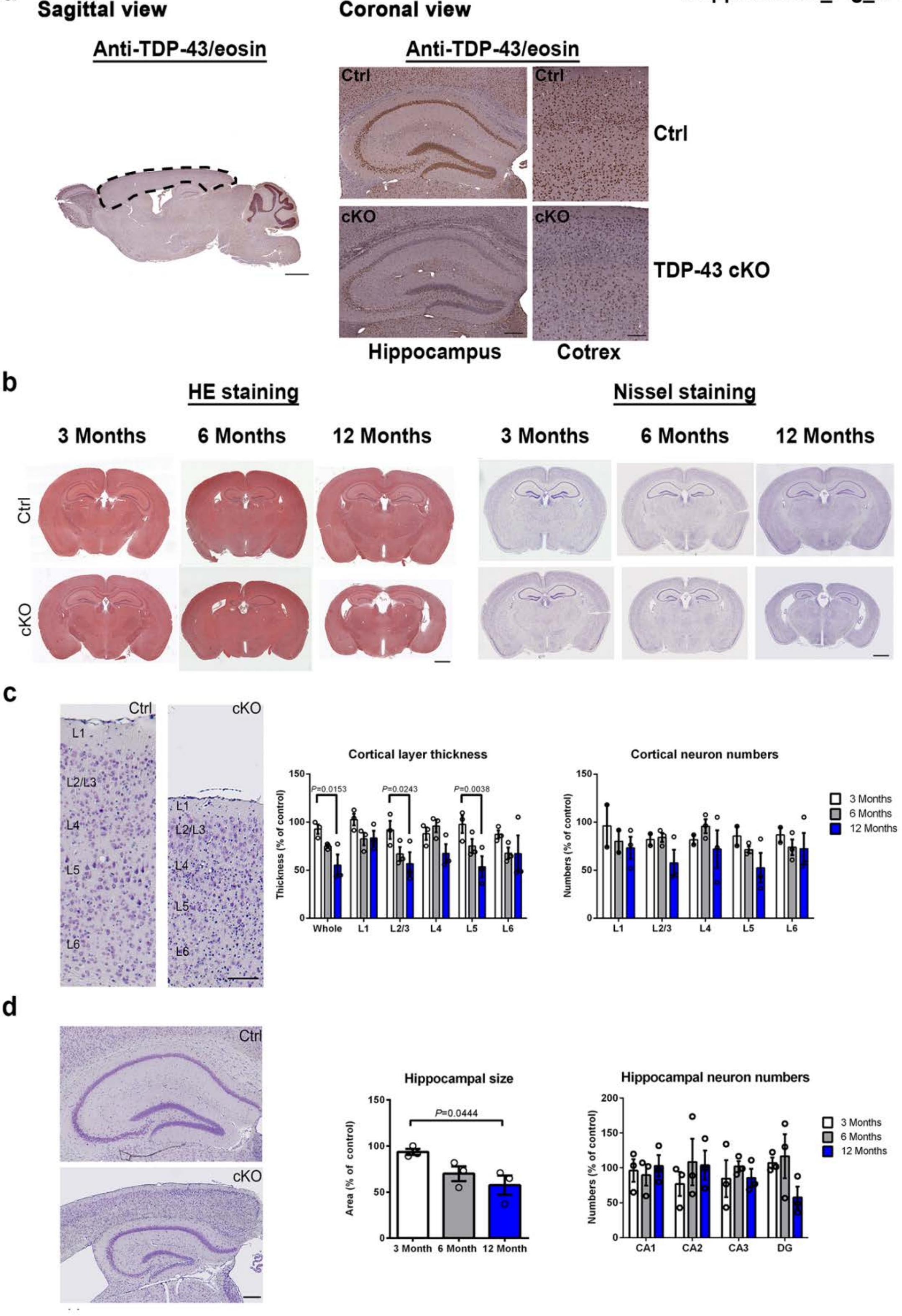

Supplemental Figure 2. Immunohistochemistry staining of brain slices. a, Representative photograph of sagittal section of the brain of 3 month-old TDP-43 cKO mice (left) and coronal sections (right panels) of 3 month-old TDP-43 cKO and Ctrl mice co stained with anti-TDP-43 and eosin counter-stain with the nucleus. The dotted line on the sagittal section indicates the neocortex region from where the RNA was isolated for RNA-Seq. **b,** Left panels, hematoxylin and eosin staining patterns. Representative photographs of stained brain sections of Ctrls (upper panels) and TDP-43 cKO mice (lower panels) at different ages are shown. Right panels, nissl staining. The representative photographs show the marked cortical atrophy and ventricle enlargement of 12 month-old of TDP-43 cKO mice in comparison with Ctrl. **c,** Left 2 panels, magnified photos of the cortical layers of the Nessiel stained brain sections of 12 month-old mice. Middle panel, histogram showing the quantification of the thickness of the different cortical layer in different groups of mice. Right panel, histogram showing the quantification of the neuron density in cortical layers of different groups of mice. Note the significant decrease of the cortical thickness of cortical layers Ⅱ/Ⅲ and Ⅴ of TDP-43 cKO mice at the age of 12 months. **d,** Left panels, magnified photos of hippocampus from the right panels in **b** above. Middle panel, histogram showing the comparison of the hippocampal sizes. Right panel, histogram showing the comparison of the neuron density in the hippocampus. Note

the significantly reduced overall hippocampal size in the brain of 12 month-old of TDP-43 cKO mice. The results were from replicated numbers of two cohorts of mice. The enlarged images were taken at 40× magnification. Two-way ANOVA was used for analysis of data in **c** and **d**, $P<0.05$ was considered significant. Scale bars, 1000 μm in a and b, 100 μm in c, 200 μm in d.

Supplemental_Fig_S3

a

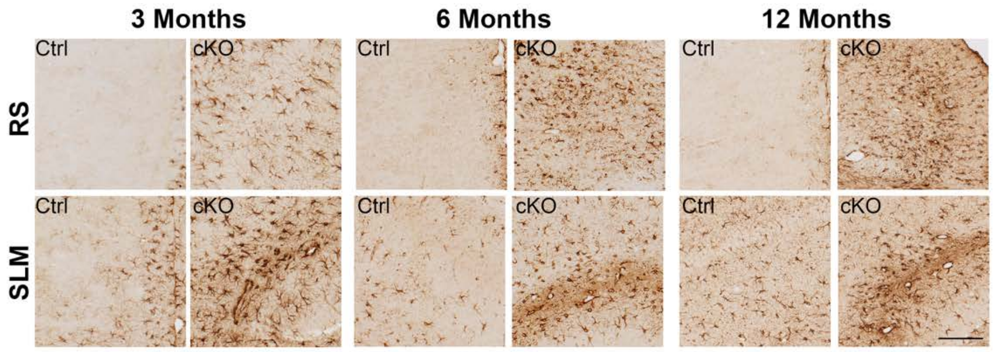

b

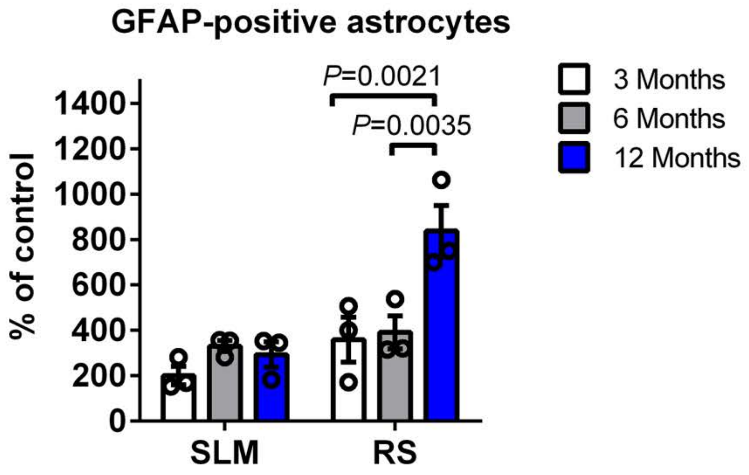

c

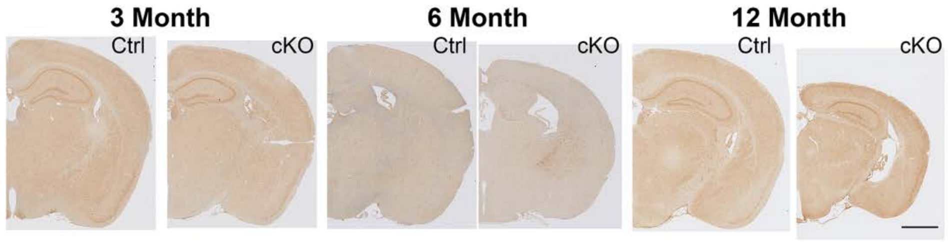

Supplemental_Figure_S3. Persisting reactive astrocytosis in TDP-43 cKO mouse forebrain. Immunohistochemistry staining with anti-GFAP revealing high numbers of GFAP-positive astrocytes in the retrosplenial cortex (RS) region of the cortex and stratum lacunosum-moleculare (SLM) region in the brain of TDP-43 cKO mice. **a**, Magnified pictures of the RS and SLM regions in **Figure 3d**. Active astrocytosis was evident for the 3 month-, 6 month-, and 12 month-old TDP-43 cKO mice in comparison to Ctrls. Note the typical tufted appearance of reactive GFAP-positive astrocytes in TDP-43 cKO mice but not in Ctrls. **b,** Quantitative comparison the GFAP-positive (+) astrocytes in SLM region of hippocampus and RS region of cortex at different ages of the mice. Note the significant increase of GFAP-positive astrocytes in 12 month-old TDP-43 cKO mice compared to Ctrls. Statistical analysis was done by unpaired t test (N=3/group) with error bars reported as SEM, $P<0.05$ was considered significant. **c**, Immunostaining of the Ctrl and TDP-43 cKO mouse brain sections with use of antibody against the microglia-specific marker Iba1. Scale bars, 1000μm.

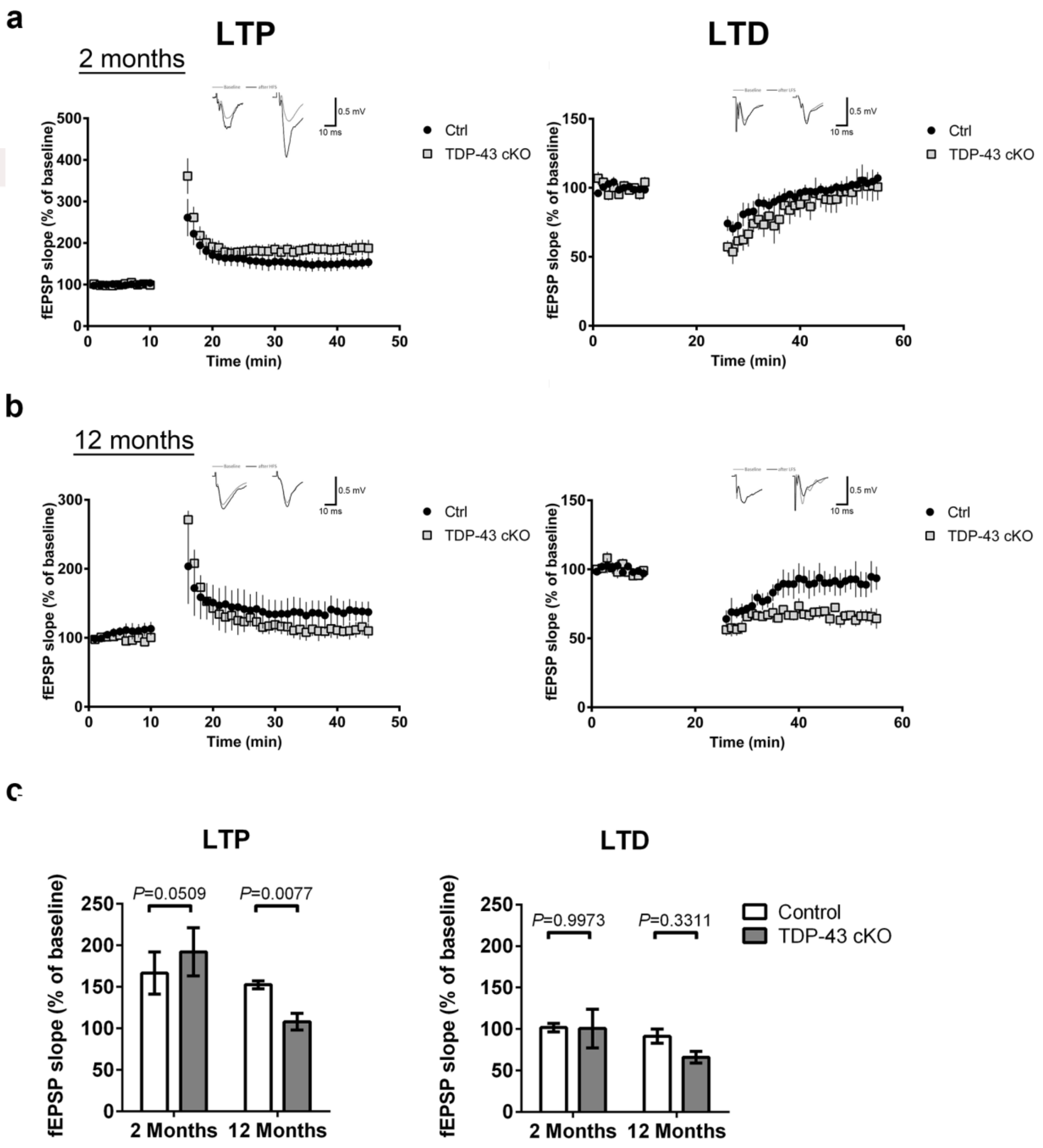

Supplemental Figure 4. Electrophysiology measurements. The field excitatory postsynaptic potential (fEPSP) was recorded at Schaffer collateral branches in CA1 region of Ctrl and TDP-43 cKO mice at the age of 2 months **(a)** and 12 months **(b)**. Upper panels: representative field responses evoked by stimulation of the hippocampal slices. The slope of fEPSP of LTP was altered in TDP-43 cKO mice at 12 month-old. **c,** Quantification results showing the average slopes of fEPSP during

the last 5 minutes of recording. N=6 animals per genotype. Statistical analysis was done by paired t test with error bars reported as SEM, $P<0.05$ was considered significant.

Supplemental_Fig_S5

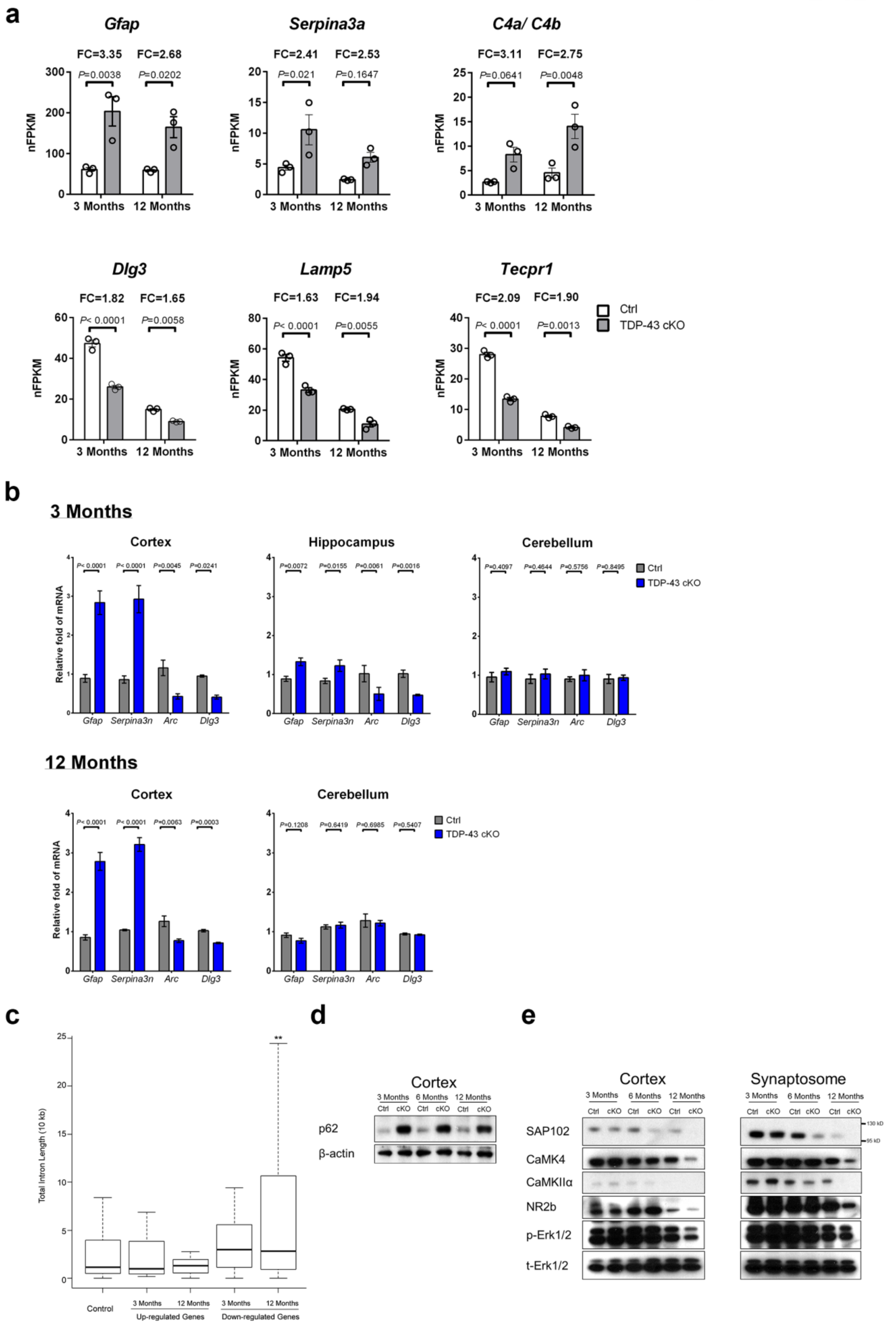

Supplemental Figure 5. Mis-regulated genes in TDP-43 cKO mice. a, Plots showing the relative changes of the expression levels of 3 inflammation-associated genes (*Gfap/ Serpina3a/C4a/C4b*), 1 synaptic function-associated gene (*Dlg3*), and 2 autophagosome/lysosome-associated genes (*Lamp5*, *Tecpr1*) in the cortex of 3 month-old and 12 month-old TDP-43 cKO mice compared to the Ctrls. N=3 mice for each group. Each data point is represented by mean±SEM. One-way ANOVA was used for analysis of data in **a**, FC, fold of change. **b,** The alterations of RNA expression of 4 genes in the cortex of 3 month- (upper panels) and 12 months-old (lower panels) TDP-43 cKO mice, as revealed by RNA-Seq data, were verified by qRT-PCR and compared among different tissues. The relative levels of the altered RNAs are shown in the histograms. N=6 for each group. Statistical analysis by unpaired t test with error bars represented as SEM. **c,** Comparisons of the intron lengths of the differentially expressed genes in the cortex of TDP-43 cKO mice. Control gene set included 100 genes, which were not differentially expressed genes and randomly selected from the mouse annotated protein coding genes. Statistical significances were evaluated using two-sided Wilcoxon rank-sum test. **P<0.01. Western blotting analysis of different proteins in the cortex and synpatosomes. The levels of the autophagy substrate p62 **(d)** and different synaptic proteins **(e)** in the cortex and /or synaptosomes of TDP-43 cKO and Ctrl mice of different ages were

compared by Western blotting. Note the significant increase of p62 in the cortex of TDP-43 cKO mice at all ages analyzed. Also, at the age of 3 months, the levels of SAP102, CaMK4, CaMK Ⅱ α, NR2b, and pErk 1/2 proteins were relatively unchanged in the cortex as well as the synpatosome of 3 month-old TDP-43 cKO mice when compared to the Ctrls, but they become significantly lower at the age of 12 months.

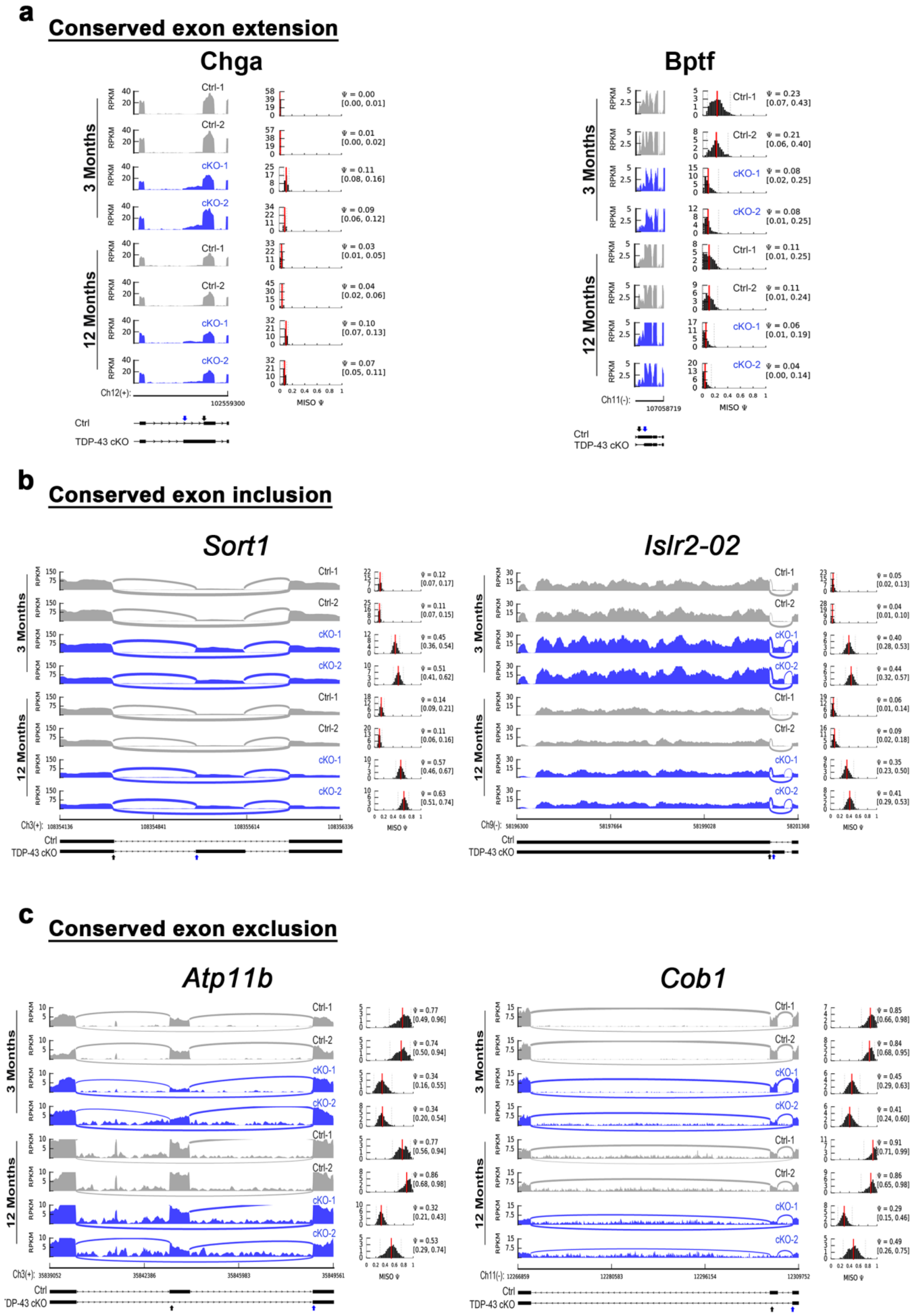

a
Conserved exon extension
Chga
Bptf
3 Months
12 Months
Ctrl-1
Ctrl-2
cKO-1
cKO-2
RPKM
MISO Ψ
Ch12(+):
102559300
Ch11(-):
107058719
Ctrl
TDP-43 cKO
b
Conserved exon inclusion
Sort1
Islr2-02
Ch3(+):
Ch9(-):
c
Conserved exon exclusion
Atp11b
Cob1
Ch11(-):

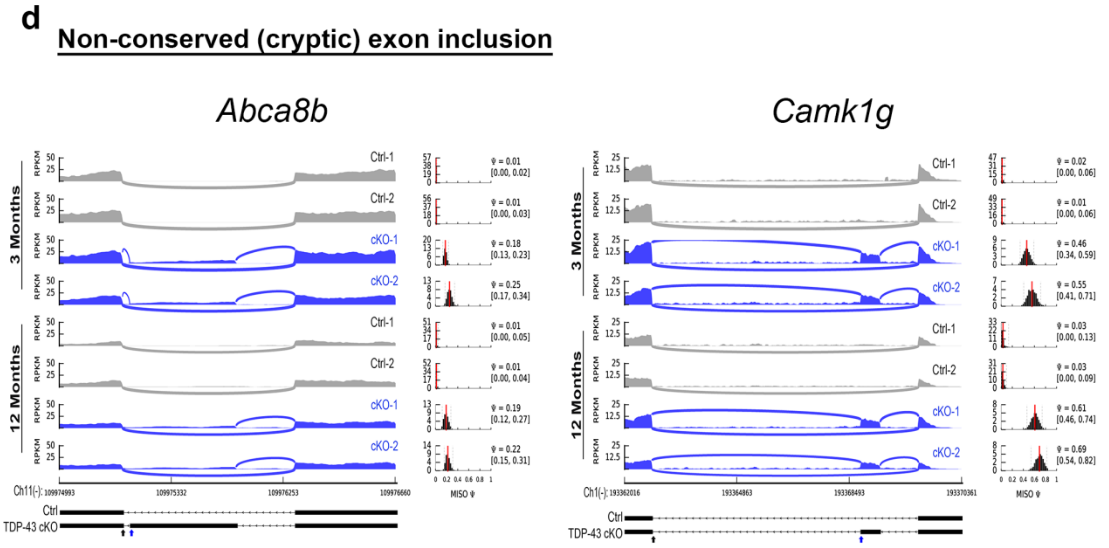

Supplemental Figure 6. Alternations of the processing events of cortical RNAs in TDP-43 cKO mice. Cortical RNAs of TDP-43 cKO and Ctrl mice at the age of 3 months or 12 months were analyzed by Cufflink/MISO as described. The RNA-seq junction reads are exemplified in **(a)** for the extension of conserved exons, in **(b)** for the inclusions and **(c)** exclusion of conserved exons, and in **(d)** for the inclusion of cryptic exons. The inclusion events of the cryptic exons occurred in the cortex of TDP-43 cKO mice with significantly higher frequencies than the Ctrl mice at 3 months as well as 12 months of age. The peaks represent the detected splicing junctions with height(s) indicating the average size factor-normalized coverage(s) within each group. The RNA processing patterns are significantly different between the TDP-43 cKO mice and Ctrls. The patterns of 2 mice of each group are compared.

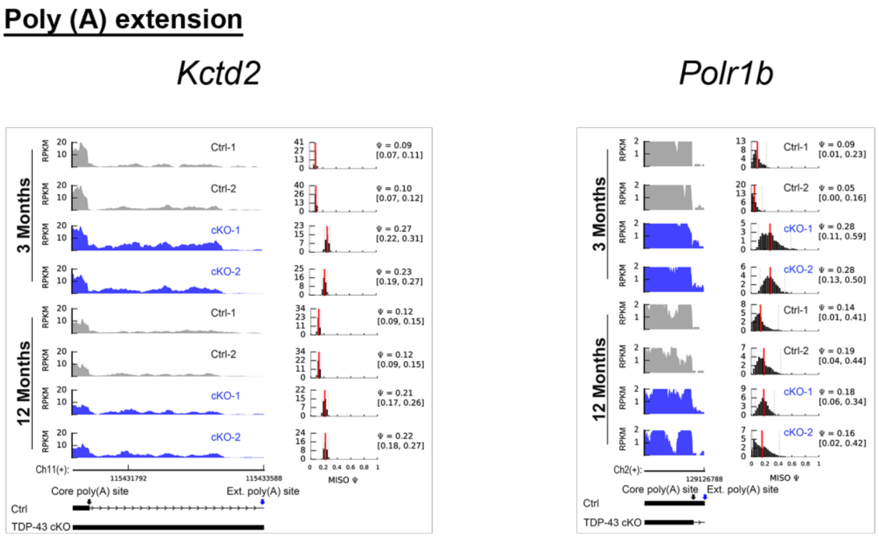

Supplemental Figure 7. The alternative uses of poly(A) sites in the cortical RNAs of TDP-43 cKO mice. The patterns of RNA-seq junction reads in the cortical transcripts with alternative poly(A) site usage are exemplified for 2 genes. The patterns of 2 mice of each group are compared.

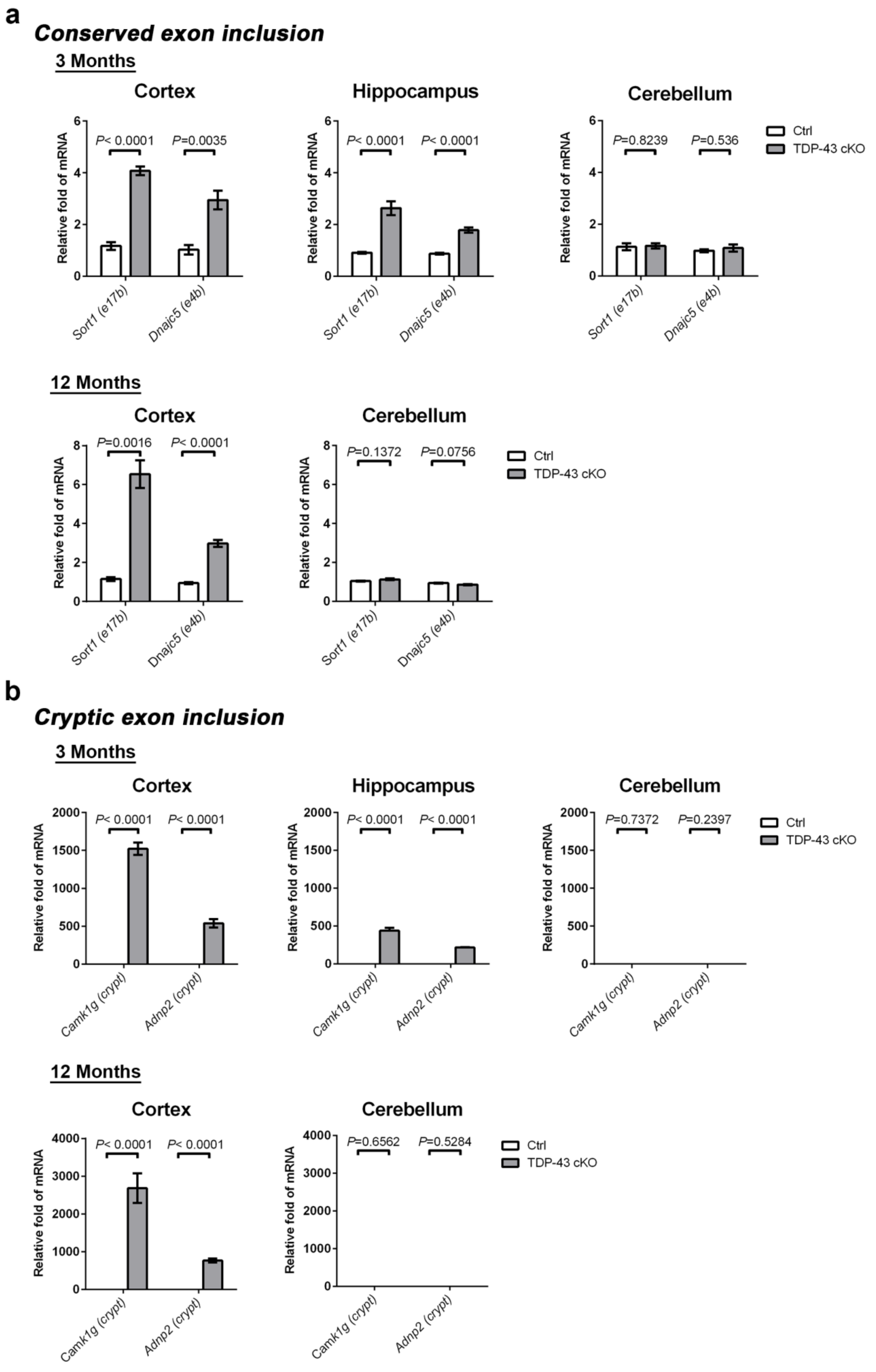
a
Conserved exon inclusion
3 Months
Cortex
Hippocampus
Cerebellum
Relative fold of mRNA
P< 0.0001
P=0.0035
P< 0.0001
P< 0.0001
P=0.8239
P=0.536
Ctrl
TDP-43 cKO
Sort1 (e17b)
Dnajc5 (e4b)
12 Months
Cortex
Cerebellum
P=0.0016
P< 0.0001
P=0.1372
P=0.0756
b
Cryptic exon inclusion
3 Months
Cortex
Hippocampus
Cerebellum
P< 0.0001
P< 0.0001
P< 0.0001
P< 0.0001
P=0.7372
P=0.2397
Camk1g (crypt)
Adnp2 (crypt)
12 Months
Cortex
Cerebellum
P< 0.0001
P< 0.0001
P=0.6562
P=0.5284

Supplemental Figure 8. qRT-PCR validation of RNA splicing events altered in 3 month- and 12 month-old TDP-43 cKO mice. The alterations of RNA splicing of 4 genes in the cortex of 3 month- and 12 month-old TDP-43 cKO mice, as revealed by RNA-seq data, were verified by qRT-PCR and compared among the cortex, hippocampus, and cerebellum. The relative levels of the altered RNAs with conserved exon inclusion **(a)** or non-conserved (cryptic) exon inclusion **(b)** are shown in the histograms. N=6 for each group. Statistical analysis was done by unpaired t test with error bars represented as SEM. $P<0.05$ was considered significant.

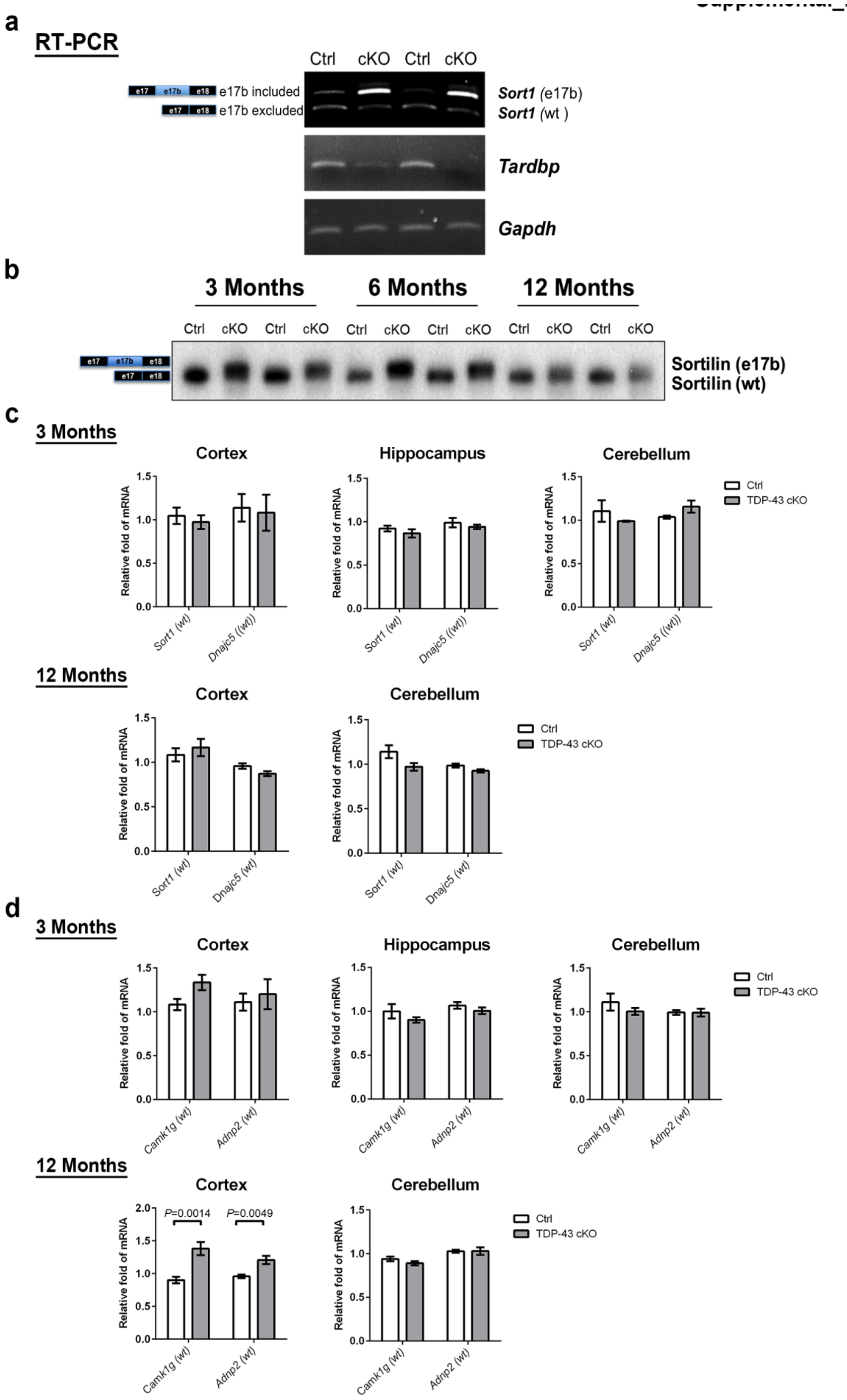
a
RT-PCR
Ctrl cKO Ctrl cKO
e17 e17b e18 e17b included
e17 e18 e17b excluded
Sort1 (e17b)
Sort1 (wt)
Tardbp
Gapdh
b
3 Months
6 Months
12 Months
Ctrl cKO Ctrl cKO Ctrl cKO Ctrl cKO Ctrl cKO Ctrl cKO
Sortilin (e17b)
Sortilin (wt)
c
3 Months
Cortex
Hippocampus
Cerebellum
Relative fold of mRNA
Sort1 (wt)
Dnajc5 ((wt))
Ctrl
TDP-43 cKO
12 Months
Cortex
Cerebellum
Sort1 (wt)
Dnajc5 (wt)
d
3 Months
Cortex
Hippocampus
Cerebellum
Camk1g (wt)
Adnp2 (wt)
12 Months
Cortex
Cerebellum
P=0.0014
P=0.0049

Supplemental Figure 9. Validation of altered splicing events in TDP-43 cKO mice.

Semi-quantitative RT-PCR **(a)** and Western blotting **(b)** were used to verify the exon 17b inclusion of *Sort1* transcript in the cortex of TDP-43 cKO mice. Samples from two mice each of 12-month-old TDP-43 cKO mice and Ctrl mice were analyzed in **(a)**, and two mice each of the TDP-43 cKO v.s. Ctrl mice at the ages of 3 months, 6 months, and 12 months were analyzed in **(b)**. qRT-PCR was used to analyze in **(c).** The relative levels of the wild type mRNAs of *Sort1* and *Dnajc5* in cortex, hippocampus, and cerebellum of 3 month- and 12 month-old TDP-43 cKO and Ctrl mice, respectively, and **(d)** the relative levels of the wild type mRNAs of *Camk1g* and *Adnp2* in cortex, hippocampus, and cerebellum of 3 month- and 12 month-old TDP-43 cKO and Ctrl mice. N=6 animals per genotype. The bars represent mean±SEM. Unpaired t test was used for analysis.

a **Calcium signaling**

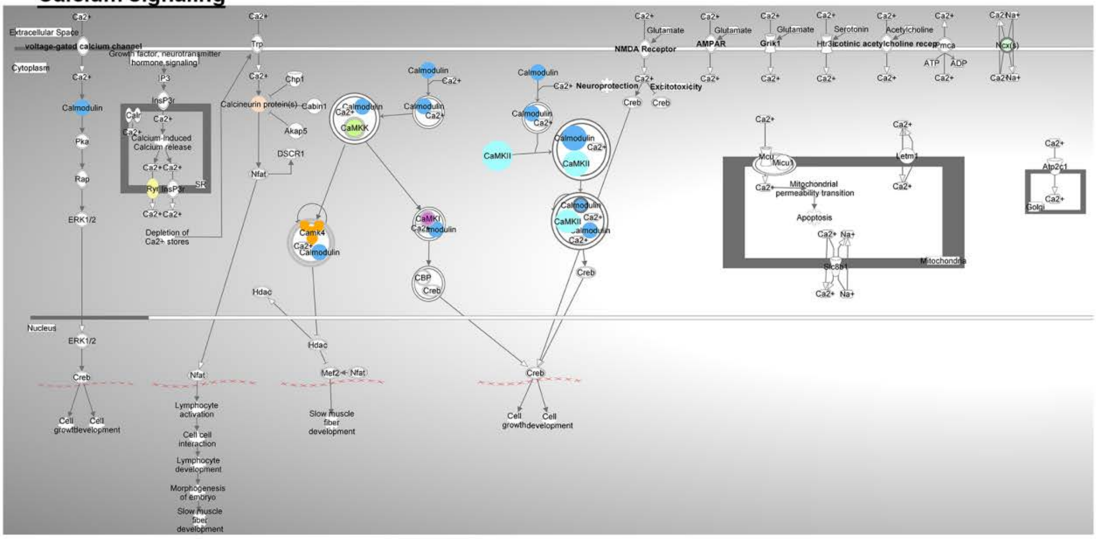

b **Synaptic long term potentiation**

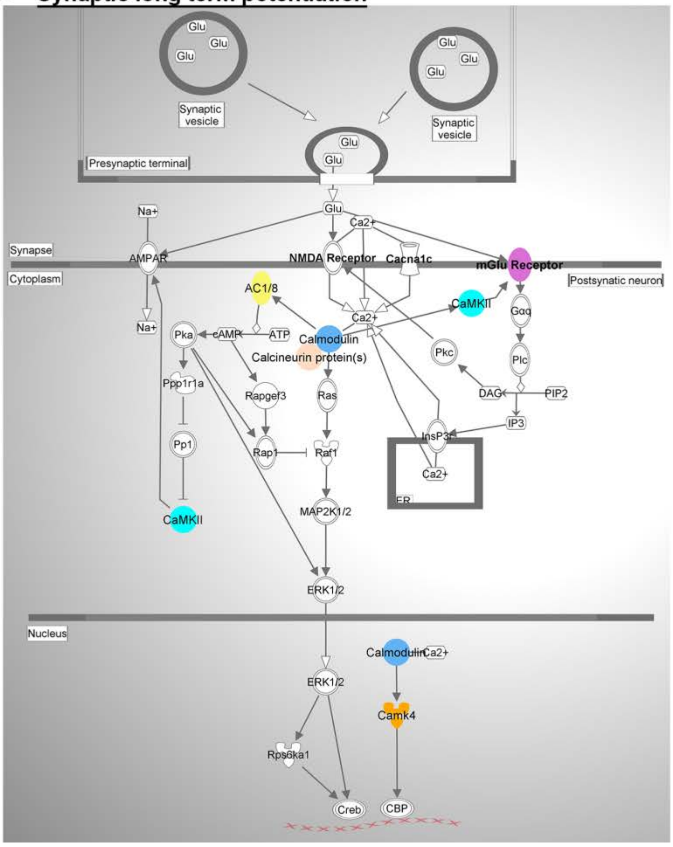

Supplemental Figure 10. Calcium signaling and synaptic long term potentiation pathway analysis using Ingenuity Pathway Analysis (IPA). IPA predicts the upstream and downstream effects of the mapped genes on **(a)** calcium and **(b)** **s**ynaptic LTP signaling pathway and hypothesizes the overall states of this pathway. Color symbols indicate genes up- and down-regulated in TDP-43 cKO mice.
