# Supplementary material for: Transcriptomopathies of pre- and post-symptomatic frontotemporal dementia-like mice with TDP-43 depletion in forebrain neurons
Source: Acta Neuropathol Commun. 2019 Mar 29;7:50. doi: 10.1186/s40478-019-0674-x (PMC6440020; doi:10.1186/s40478-019-0674-x)
Supplement: Supplementary file 2 — Table S1. List of the chromosome numbers, donor positions, acceptors positions and gene names of the transcripts from which the individual circularRNAs change in the neocortex of 3- and 12-month-old TDP-43 cKO mice, but not Ctrl mice are listed. (PDF 141 kb) [file 40478_2019_674_MOESM2_ESM.pdf]

Supplemental Table 1. <u>**List of the chromosome numbers, donor positions, acceptors positions and gene names of the transcripts from which the individual circular RNAs change in the cortex of 3 month and 12 month-old TDP-43 cKO mice, but not Ctrl mice are listed.**</u>

			CircRNA expression				Significance		Congate gene expression		Significance	
			3 Months		12 Months		3 Months	12 Months	3 Months	12 Months	3 Months	12 Months
			log_2 FC		log_2 FC				log_2 FC	log_2 FC		
ID	Strand	Gene	DESeq	edgeR	DESeq	edgeR	$p<0.05$	$p<0.05$			$p<0.05$	$p<0.05$
chr7:99935162_chr7:99955540	-	Rnf169	7.46	8.87	0.45	0.53	●		0.00	-0.75		●
chr19:4792398_chr19:4794009	-	Gm21992	7.40	8.80	-0.06	0.01	●		0.00	-0.75		●
chr15:39616510_chr15:39566943	+	Rims2	7.25	8.62	-0.14	-0.06	●		-0.31	-0.39		●
chr2:68390914_chr2:68410141	-	Stk39	6.33	7.62	-0.29	-0.23	●		-0.21	-0.30		●
chr2:25965663_chr2:25966948	-	Camsap1	5.82	7.17	-0.09	-0.01	●		-0.36	-0.36		●
chr8:79136663_chr8:79118175	+	Zfp827	5.67	6.91	-0.39	-0.32	●		-0.33	-0.40		●
chr8:77344640_chr8:77365160	-	Arhgap10	5.65	6.93	0.00	0.00	●		-0.05	0.02		
chr6:84989325_chr6:85005036	-	Exoc6b	5.32	6.57	0.36	0.47	●		-0.09	-0.27		
chr11:84195687_chr11:84193079	+	Acaca	4.96	6.17	0.37	0.45	●		-0.20	-0.19		
chr12:99388361_chr12:99388917	-	Foxn3	4.87	6.06	-2.09	-3.98	●		-0.20	0.00		
chr2:37627435_chr2:37647285	-	Strbp	4.84	6.06	1.01	1.08	●		0.08	0.02		
chr2:70750435_chr2:70786995	-	Tlk1	4.83	6.01	-1.46	-3.44	●		0.36	0.24		
chr4:109083089_chr4:109087485	-	Osbpl9	4.69	5.95	0.00	0.00	●		-0.20	-0.18		
chr17:42845326_chr17:42852528	-	Cd2ap	4.65	5.89	0.39	0.49	●		-0.05	0.00		
chr12:77332329_chr12:77323845	+	Fut8	4.34	5.62	-0.05	0.00	●		0.17	0.13		
chr12:100446860_chr12:100495440	-	Ttc7b	4.29	5.54	0.62	0.76	●		0.07	0.03		
chr19:29753600_chr19:29755017	-	30021J03R	4.28	5.50	0.67	0.78	●		0.10	-0.02		
chr3:116645366_chr3:116651499	-	43191,Mfs	4.26	5.46	0.06	0.13	●		-0.03	-0.22		
chr1:8554725_chr1:8624881	-	Sntg1	4.12	5.38	-1.54	-1.49	●		-0.13	-0.34		
chr19:7026292_chr19:7032649	-	Stip1	4.11	5.29	-0.19	-0.10	●		-0.10	-0.09		
chr4:129178241_chr4:129183717	-	S100pbp	4.07	5.29	0.34	0.41	●		0.09	0.11		
chr3:103311513_chr3:103310309	+	Trim33	4.07	5.30	-0.26	-0.20	●		-0.18	0.00		
chr11:80227332_chr11:80223903	+	Rhot1	4.04	5.36	-0.21	-0.08	●		-0.07	-0.08		
chry:1186774_chry:1199513	-	Uty	4.01	5.34	0.54	0.65	●		0.00	-0.21		
chr2:169886459_chr2:169883526	+	Tshz2	3.98	5.19	-3.38	-4.94	●		-0.05	-0.24		
chr9:8658377_chr9:8634047	+	Trpc6	-1.05	-1.30	-0.23	-0.15	●		-0.60	-0.56	●	●
chr4:132673032_chr4:132656693	+	Eya3	-3.94	-5.52	-0.16	-0.08	●		-0.12	-0.06		
chr17:25235182_chr17:25235627	-	Gnptg	-4.00	-5.61	0.25	0.30	●		0.00	-0.04		
chr18:77150098_chr18:77133091	+	Pias2	-4.22	-5.77	0.42	0.53	●		0.04	0.03		
chr13:106812208_chr13:106848336	-	Ipo11	-4.29	-5.84	-0.41	-0.31	●		0.00	0.00		
chr17:88463263_chr17:88462713	+	Foxn2	-4.39	-5.97	0.37	0.46	●		-0.04	0.03		
chr8:77382725_chr8:77413627	-	Arhgap10	-4.60	-6.16	-0.63	-0.56	●		-0.16	-0.06		
chr15:81382980_chr15:81392322	-	St13	-4.60	-6.16	-0.20	-0.11	●		-0.09	-0.11		
chr8:67904117_chr8:67908889	-	Psd3	-4.72	-6.27	0.40	0.48	●		0.00	0.00		
chr2:140042094_chr2:140057499	-	Tasp1	-5.00	-6.55	0.49	0.59	●		-0.06	-0.14		
chr15:41826052_chr15:41797474	+	Oxr1	-5.22	-6.79	-0.61	-0.51	●		-0.27	-0.07		
chr7:141992300_chr7:141987751	+	Brsk2	-6.00	-7.61	0.05	0.11	●		-0.22	-0.28		
chr7:99935162_chr7:99943105	-	Rnf169	-6.29	-6.98	-0.47	-0.46	●		-0.22	-0.28		
chr9:96591950_chr9:96611499	-	Rasa2	-7.32	-9.06	-0.21	-0.13	●		-0.21	-0.30		
chr9:32208265_chr9:32181842	+	Arhgap32	5.33	6.53	6.11	7.69	●	●	-0.39	0.00	●	
chr3:50417988_chr3:50438915	-	Slc7a11	-3.95	-5.52	5.06	6.56	●	●	0.44	0.42	●	●
chr19:57797503_chr19:57751630	+	Atrnl1	-5.58	-7.17	6.29	7.88	●	●	-0.22	-0.37		●
chr9:77164765_chr9:77190737	-	Mlip	5.10	6.31	-6.44	-8.13	●	●	-0.36	-0.71		●
chr16:17373350_chr16:17389457	-	Pi4ka	6.23	7.50	1.07	1.15	●	●	-0.11	-0.18		
chr13:45735115_chr13:45956575	-	Atxn1	-5.64	-7.23	6.39	8.00	●	●	-0.05	-0.34		
chr17:74241905_chr17:74258505	-	Memo1	3.95	5.27	-4.50	-6.11	●	●	0.11	0.00		
chr2:33260478_chr2:33324703	-	Ralgps1	6.11	7.40	-6.39	-8.09	●	●	-0.18	-0.20		
chr9:59426123_chr9:59454469	-	Arih1	4.98	6.17	-5.47	-7.07	●	●	-0.09	-0.01		
chr1:66801049_chr1:66802168	-	Kansl1l	4.09	5.29	2.73	2.89	●	●	1.46	0.00		
chr11:97531804_chr11:97535827	-	Srcin1	5.32	6.57	5.88	7.45	●	●	0.22	0.25		
chr11:104423922_chr11:104426468	-	Kansl1	-4.21	-5.77	-4.29	-5.90	●	●	-0.25	-0.05		
chr13:91642391_chr13:91524585	+	Ssbp2	4.17	5.38	5.12	6.60	●	●	0.55	-0.19		
chr14:12418005_chr14:12457829	-	Cadps	-4.86	-6.42	-5.86	-7.50	●	●	-0.13	-0.13		
chr14:30484586_chr14:30482193	+	Dcp1a	1.78	1.54	1.68	1.79	●	●	0.26	0.17		
chr16:11116795_chr16:11128703	-	Txndc11	4.75	5.95	6.08	7.66	●	●	0.00	-0.21		
chr16:17373350_chr16:17378514	-	Pi4ka	3.95	5.19	3.52	3.95	●	●	-0.11	-0.18		
chr3:51299783_chr3:51326035	-	Elf2	4.05	5.37	5.61	7.14	●	●	-0.34	-0.38		
chr4:117615241_chr4:117582600	+	Eri3	-4.35	-5.91	-5.09	-6.70	●	●	-0.08	-0.14		
chr5:23540375_chr5:23565265	-	Srpk2	4.10	5.29	4.30	5.85	●	●	-0.12	-0.13		
chr5:143707549_chr5:143686473	+	Cyth3	4.37	5.55	5.33	6.87	●	●	0.19	0.08		
chr9:24462577_chr9:24485729	-	Dpy19l1	-3.96	-5.53	-4.66	-6.23	●	●	-0.16	-0.20		
chr13:11750689_chr13:11769999	-	Ryr2	0.85	2.60	3.92	5.47		●	-0.38	0.00	●	
chr1:181109160_chr1:181123984	-	Nvl	2.45	2.36	2.16	2.30		●	0.77	0.06	●	
chr5:120655129_chr5:120654806	+	Rasal1	2.35	4.02	2.28	2.42		●	-0.39	-0.52	●	●
chr6:140563648_chr6:140543720	+	Plekha5	0.40	0.20	-3.90	-5.43		●	-0.47	-0.36	●	●
chr8:86860426_chr8:86862110	-	N4bp1	1.22	0.97	1.41	1.52		●	0.59	0.42	●	●
chr15:82825231_chr15:82857284	-	Tcf20	0.00	0.00	-5.20	-6.78		●	-0.44	-0.40	●	●
chr1:140450304_chr1:140362949	+	Kcnt2	-1.00	-1.26	-0.69	-0.62		●	-0.54	-0.89	●	●
chr1:140484233_chr1:140362949	+	Kcnt2	0.00	0.00	4.26	5.84		●	-0.54	-0.89	●	●
chr8:77384751_chr8:77413627	-	Arhgap10	3.35	4.72	-4.08	-5.65		●	0.00	-0.75		●
chr18:33129833_chr18:33077993	+	Camk4	-0.44	-0.67	5.21	6.70		●	-0.24	-0.73		●
chr8:77310731_chr8:77358634	-	Arhgap10	-3.85	-5.43	4.62	6.14		●	0.00	-0.75		●
chr8:77310731_chr8:77365160	-	Arhgap10	-0.86	-1.11	5.29	6.83		●	0.00	-0.75		●
chr3:89007877_chr3:89001773	+	Ash1l	0.00	0.00	-5.25	-6.82		●	-0.25	-0.34		●

chr5:73534697_chr5:73520116	+	Dcun1d4	0.13	-0.08	4.25	6.35		●	-0.26	-0.36		●
chr7:14578736_chr7:14609339	-	Nlrp5-ps	0.00	0.00	-5.30	-6.92		●	0.00	-1.07		●
chr9:77164765_chr9:77217048	-	Mlip	-0.85	-1.11	6.56	8.22		●	-0.36	-0.71		●
chr12:3648389_chr12:3617606	+	Dtnb	0.13	-0.11	-8.83	-10.83		●	-0.25	-0.43		●
chr12:3686862_chr12:3617606	+	Dtnb	-0.28	-0.55	-6.59	-8.30		●	-0.25	-0.43		●
chr19:57777945_chr19:57751630	+	Atrnl1	0.03	-0.21	-6.64	-8.36		●	-0.22	-0.37		●
chr5:73534697_chr5:73510886	+	Dcun1d4	-0.80	-1.04	-6.81	-8.54		●	-0.26	-0.36		●
chr5:73544155_chr5:73520116	+	Dcun1d4	-0.32	-0.53	-4.76	-6.33		●	-0.26	-0.36		●
chr7:23438059_chr7:14609339	-	Nlrp5-ps	0.00	0.00	5.77	7.31		●	0.00	-1.07		●
chr7:97894013_chr7:97883472	+	Pak1	3.84	5.17	-4.35	-5.96		●	-0.29	-0.36		●
chr13:35816575_chr13:35790101	+	Cdyl	-3.82	-5.43	4.99	6.51		●	-0.30	-0.44		●
chr10:127454756_chr10:127452692	+	R3hdm2	0.00	0.00	3.97	5.48		●	-0.20	-0.43		●
chr12:38190134_chr12:38147503	+	Dgkb	-1.00	-3.24	-3.80	-5.41		●	-0.14	-0.37		●
chr15:39517871_chr15:39452224	+	Rims2	2.04	4.21	-3.94	-5.50		●	-0.21	-0.30		●
chr2:24631899_chr2:24651218	-	Cacna1b	2.08	3.78	4.09	5.63		●	-0.13	0.40		●
chr2:68307041_chr2:68359034	-	Stk39	1.07	0.87	4.98	6.46		●	-0.33	-0.40		●
chr12:3718489_chr12:3617606	+	Dtnb	0.42	1.82	-2.85	-2.91		●	-0.25	-0.43		●
chr1:8624779_chr1:8682029	-	Sntg1	-0.08	-0.35	3.83	5.38		●	-0.05	-0.24		
chr1:53256629_chr1:53290423	-	Pms1	0.00	0.00	-4.04	-5.64		●	-0.21	-0.41		
chr10:9825370_chr10:9838160	-	Stxbp5	1.57	3.72	3.15	3.50		●	-0.14	-0.10		
chr10:43413547_chr10:43393871	+	Pdss2	-0.60	-0.84	-4.64	-6.18		●	-0.17	-0.20		
chr10:90163304_chr10:90069468	+	Anks1b	-2.10	-4.16	-3.91	-5.50		●	-0.26	-0.12		
chr12:24939348_chr12:24878191	+	Mboat2	0.00	0.00	4.30	5.74		●	-0.22	-0.25		
chr12:50419853_chr12:50428234	-	Prkd1	1.09	3.11	3.86	5.46		●	0.04	0.02		
chr12:73219142_chr12:73179205	+	Mnat1	0.00	0.00	4.09	5.58		●	0.02	-0.09		
chr13:59495333_chr13:59504310	-	Agtpbp1	-0.36	-0.68	-3.84	-5.35		●	-0.26	-0.26		
chr14:12411554_chr14:12440915	-	Cadps	-0.39	-0.62	4.09	5.64		●	-0.13	-0.13		
chr14:12411554_chr14:12457829	-	Cadps	2.96	4.41	4.25	5.73		●	-0.13	-0.13		
chr14:28141381_chr14:28029434	+	Erc2	0.85	2.60	4.41	5.88		●	-0.17	0.00		
chr14:28141381_chr14:28040343	+	Erc2	-0.32	-0.55	-1.75	-1.69		●	-0.17	0.00		
chr14:57856610_chr14:57878459	-	Zdhhc20	1.91	3.77	-4.07	-5.65		●	0.11	-0.01		
chr15:69049461_chr15:69013358	+	Khdrbs3	0.00	0.00	-3.88	-5.50		●	-0.16	-0.25		
chr16:17381801_chr16:17389457	-	Pi4ka	3.71	4.98	2.08	2.19		●	-0.11	-0.18		
chr16:37236230_chr16:37291063	-	Stxbp5l	3.25	4.57	4.59	6.08		●	-0.02	-0.07		
chr18:23597558_chr18:23583084	+	Dtna	0.86	0.61	4.59	6.05		●	-0.10	-0.09		
chr19:16759587_chr19:16766540	-	Vps13a	1.46	3.49	4.14	5.63		●	-0.21	-0.22		
chr2:25937946_chr2:25941469	-	Camsap1	0.00	0.00	4.01	5.48		●	-0.16	-0.06		
chr2:37509078_chr2:37469377	+	Rabgap1	0.57	0.35	3.88	5.38		●	0.01	0.02		
chr2:61627839_chr2:61613651	+	Tank	1.95	4.00	4.09	5.65		●	0.00	0.02		
chr2:91825352_chr2:91805024	+	Ambra1	-0.20	-0.42	-4.97	-6.53		●	-0.02	-0.12		
chr2:94371737_chr2:94378858	-	Ttc17	3.30	4.72	4.99	6.52		●	0.14	0.11		
chr2:131287597_chr2:131273911	+	Pank2	-0.23	-0.49	-4.26	-5.85		●	0.01	-0.12		
chr2:139996073_chr2:140057499	-	Tasp1	-0.61	-0.89	-5.28	-6.90		●	-0.13	-0.34		
chr3:88908789_chr3:88901578	+	Gon4l	1.74	3.77	3.87	5.46		●	0.08	0.03		
chr3:129996681_chr3:130005096	-	Sec24b	3.74	4.98	4.39	5.84		●	-0.02	0.06		
chr3:158127215_chr3:158148699	-	Lrrc7	2.70	4.23	4.01	5.51		●	-0.03	-0.15		
chr4:41189286_chr4:41183245	+	Ube2r2	3.81	5.09	4.73	6.28		●	0.04	-0.14		
chr4:123444835_chr4:123456749	-	Macf1	2.30	4.02	3.97	5.48		●	-0.11	-0.05		
chr4:126453558_chr4:126454424	-	Ago1	1.47	3.48	3.95	5.39		●	0.06	-0.15		
chr5:53199901_chr5:53200471	-	Sel1l3	1.35	1.12	1.79	1.89		●	0.14	-0.19		
chr5:90974415_chr5:90946790	+	Mthfd2l	0.89	0.69	4.86	6.41		●	-0.14	-0.22		
chr5:110753804_chr5:110756766	-	Ep400	0.00	0.00	-4.12	-5.66		●	-0.10	-0.12		
chr6:36894776_chr6:36975247	-	Dgki	0.00	0.00	-4.14	-5.66		●	-0.16	-0.17		
chr6:37048120_chr6:37087694	-	Dgki	-0.74	-1.00	-4.05	-5.91		●	-0.16	-0.17		
chr7:46515668_chr7:46635556	-	Sergef	0.83	0.66	-3.83	-5.37		●	-0.16	-0.32		
chr7:132771578_chr7:132779385	-	Fam53b	0.44	0.19	1.34	1.43		●	0.00	0.00		
chr7:137453614_chr7:137444979	+	Glrx3	3.40	4.75	-3.80	-5.42		●	-0.11	0.00		
chr8:46818908_chr8:46793493	+	Irf2	-0.16	-0.36	4.04	5.49		●	-0.13	-0.36		
chr8:88723212_chr8:88702305	+	Cyld	-0.28	-0.49	4.06	5.57		●	-0.07	-0.07		
chr9:90103655_chr9:90110172	-	Morf4l1	0.00	0.00	4.45	5.95		●	-0.05	-0.26		
chr9:96340202_chr9:96345893	-	Atp1b3	3.63	4.97	4.24	5.73		●	0.18	-0.09		
chr10:9778021_chr10:9800068	-	Stxbp5	0.00	0.00	-4.63	-6.18		●	-0.14	-0.10		
chr10:12436281_chr10:12455564	-	Utrn	-1.18	-1.42	5.26	6.80		●	-0.10	-0.18		
chr10:90923602_chr10:90895099	+	Anks1b	-0.01	-0.26	5.13	6.69		●	-0.26	-0.12		
chr11:104350487_chr11:104378826	-	Kansl1	-0.35	-0.58	5.86	7.44		●	-0.25	-0.05		
chr12:24924294_chr12:24878191	+	Mboat2	-0.75	-0.98	7.44	9.17		●	-0.22	-0.25		
chr12:24934622_chr12:24878191	+	Mboat2	-0.40	-0.66	6.28	7.86		●	-0.22	-0.25		
chr12:81480931_chr12:81510965	-	Gm20498	0.00	0.00	-4.44	-5.97		●	0.00	0.00		
chr12:111618383_chr12:111592577	+	Mark3	0.31	0.05	4.85	6.32		●	-0.06	-0.04		
chr12:113629994_chr17:66053091	-	Ankrd12	0.00	0.00	-8.60	-10.57		●	-0.11	-0.18		
chr14:56748835_chr14:56772006	-	Pspc1	-0.24	-0.45	5.38	6.91		●	-0.13	-0.13		
chr18:63825238_chr18:63791796	+	Wdr7	0.00	0.00	-5.44	-7.04		●	-0.09	-0.02		
chr19:14516359_chr19:14564451	-	Tle4	-0.66	-0.95	5.19	6.75		●	-0.14	-0.07		
chr2:14026906_chr2:14045255	-	Hacd1	0.00	0.00	-5.00	-6.60		●	0.00	0.00		
chr3:55853771_chr3:55891674	-	Nbea	3.35	4.72	-4.44	-5.99		●	-0.15	-0.13		
chr6:31433131_chr6:31418931	+	Mkln1	0.10	-0.15	5.60	7.12		●	-0.11	-0.08		
chr6:38470222_chr6:38461475	+	Ubn2	-0.08	-0.35	5.02	6.55		●	-0.26	-0.11		
chr7:81893799_chr7:81905636	-	Hdgfrp3	0.15	-0.09	7.83	9.60		●	0.10	-0.08		
chr9:24473774_chr9:24485729	-	Dpy19l1	0.00	0.00	-5.82	-7.46		●	-0.16	-0.20		
chr9:59843138_chr9:59801914	+	Myo9a	-1.02	-1.26	5.43	7.01		●	-0.09	-0.21		
chr9:100757786_chr9:100705153	+	Stag1	-0.20	-0.45	5.01	6.52		●	-0.14	-0.29		

chr9:100738142_chr9:100663250	+	Stag1	3.70	4.97	-5.00	-6.61		●	-0.14	-0.29		
chr1:13221307_chr1:13286025	-	Ncoa2	-0.54	-0.82	-6.62	-8.33		●	-0.28	-0.13		
chr1:190871592_chr1:190899571	-	Rps6kc1	0.61	0.36	-7.37	-9.16		●	-0.21	-0.30		
chr10:18625148_chr10:18630926	-	Arfgef3	0.00	0.00	4.79	6.26		●	0.00	0.00		
chr10:89112791_chr10:89125224	-	Ano4	1.65	1.46	-4.55	-6.12		●	-0.06	-0.05		
chr12:51621780_chr12:51661713	-	Strn3	0.26	0.01	-4.45	-5.99		●	0.03	-0.06		
chr12:55782782_chr12:55795776	-	Ralgapa1	0.00	0.00	4.74	6.20		●	-0.14	-0.18		
chr12:103149081_chr12:103146302	+	Unc79	0.00	0.00	3.00	3.23		●	-0.18	-0.24		
chr13:93349297_chr13:93341756	+	Homer1	0.93	0.72	-5.20	-6.80		●	-0.03	-0.27		
chr15:4059996_chr15:4047465	+	Oxct1	-0.64	-0.89	-5.98	-7.65		●	-0.07	-0.11		
chr15:95374728_chr15:95385156	-	Nell2	0.36	0.14	-5.16	-6.78		●	-0.14	-0.15		
chr16:20657609_chr16:20656543	+	Psmd2	0.00	0.00	5.55	7.07		●	-0.06	-0.04		
chr16:33457001_chr16:33421321	+	Zfp148	-0.73	-0.99	-4.88	-6.45		●	0.01	-0.09		
chr16:94403368_chr16:94383912	+	Ttc3	-0.12	-0.36	-7.35	-9.15		●	-0.16	-0.13		
chr2:156254310_chr2:156223335	+	Phf20	0.00	0.00	4.63	6.09		●	-0.19	-0.18		
chr3:133348190_chr3:133320936	+	Ppa2	0.22	0.01	-4.92	-6.49		●	-0.26	-0.30		
chr4:9583812_chr4:9610938	-	Asph	-0.10	-0.36	-4.56	-6.13		●	-0.10	-0.16		
chr4:150477468_chr4:150439343	+	Rere	-0.16	-0.39	-5.78	-7.42		●	0.00	-0.12		
chr4:150534945_chr4:150500003	+	Rere	0.75	0.53	-3.90	-5.43		●	0.00	-0.12		
chr5:20569354_chr5:20543585	+	Magi2	0.66	0.44	-5.36	-6.96		●	-0.09	-0.05		
chr5:71006107_chr5:71034797	-	Gabra2	0.00	0.00	4.78	6.31		●	-0.15	0.25		
chr5:150604177_chr5:150608440	-	N4bp2l2	-0.39	-0.62	-5.85	-7.48		●	-0.09	-0.08		
chr6:31459067_chr6:31418931	+	Mkln1	0.00	0.00	4.76	6.26		●	-0.11	-0.08		
chr8:18689059_chr8:18641565	+	Mcph1	0.00	-0.26	-5.54	-7.17		●	0.29	0.09		
chr8:22545600_chr8:22535019	+	Slc20a2	-0.33	-0.58	-4.26	-5.85		●	0.05	-0.04		
chr9:96582743_chr9:96611499	-	Rasa2	0.00	0.00	4.84	6.31		●	0.00	-0.21		
chr9:100803354_chr9:100776756	+	Stag1	-0.68	-0.93	-4.74	-6.34		●	-0.14	-0.29		

FC, fold change. DESeq, differential gene expression analysis based on the negative binomial distribution. edgeR, differential expression analysis of digital gene expression data.
