# Supplementary material for: Transcriptomopathies of pre- and post-symptomatic frontotemporal dementia-like mice with TDP-43 depletion in forebrain neurons
Source: Acta Neuropathol Commun. 2019 Mar 29;7:50. doi: 10.1186/s40478-019-0674-x (PMC6440020; doi:10.1186/s40478-019-0674-x)
Supplement: Supplementary file 3 — Table S2. Changes of the mRNA levels of social behaviour-related genes in the neocortex of TDP-43 cKO mice relative to Ctrl mice. (PDF 72 kb) [file 40478_2019_674_MOESM3_ESM.pdf]

Supplemental Table 2. Changes of the mRNA levels of social behaviour-related genes in the cortex of TDP-43 cKO mice relative to Ctrl mice.

Gene symbol	Name	Log_2(TDP-43 cKO/ Ctrl)		Significant (q<0.05)		Anxiety-like behaviour	Social behaviour defecit
		3 Months	12 months	3 Months	12 months		
Slc6a3	solute carrier family 6 member 3	2.18824	0.796075	●		Perona et al. 2008	
Egr1	early growth response 1	-0.420573	-0.904261		●	Ko et al. 2005	
Fos	FBJ osteosarcoma oncogene	-0.765909	-0.839921		●	Blake et al. 2017	
Camk4	calcium/calmodulin-dependent protein kinase IV	-0.244258	-0.725314		●	Shum et al. 2005	
Rims1	regulating synaptic membrane exocytosis 1	-0.421464	-0.725084		●		Blundell et al. 2010
Ephb6	Eph receptor B6	-0.368624	-0.628178		●		Bianco et al. 2007
Camk2a	calcium/calmodulin-dependent protein kinase II alpha	-0.496747	-0.603486		●	Hagihara et al. 2016	
Rorb	RAR-related orphan receptor beta	-0.341338	-0.600719		●	Masana et al. 2007	
Adcy1	adenylate cyclase 1	-0.254744	-0.588347		●		Blake et al. 2017
Cadps2	Ca2+-dependent activator protein for secretion 2	-0.534627	-0.557333		●		Sadakata et al. 2007
Dlgap3	discs, large (Drosophila) homolog-associated protein 3	-0.428386	-0.518075		●	Welch et al. 2007	
Snap25	synaptosomal-associated protein 25	-0.434212	-0.503622		●	Kataoka et al. 2011	
Cplx2	complexin 2	-0.262926	-0.502105		●		Glynn et al. 2010
Ppp3r1	protein phosphatase 3, regulatory subunit B, alpha isoform (calcineurin B, type	-0.251026	-0.477824		●		Miyakawa et al. 2003
Atp1a1	ATPase, Na+/K+ transporting, alpha 1 polypeptide	-0.330546	-0.435031		●	Blake et al. 2017	
Irs4	insulin receptor substrate 4	0.220604	1.78847		●		Xu et al. 2012
Oxt	oxytocin/neurophysin I prepropeptide	1.11135	2.9496		●		Ferguson et al. 2001
